# Intellectual humility is reliably associated with constructive responses to conflict

**DOI:** 10.1371/journal.pone.0309848

**Published:** 2024-09-06

**Authors:** Jonah Koetke, Karina Schumann, Keith Welker, Peter T. Coleman

**Affiliations:** 1 Department of Psychology, University of Pittsburgh, Pittsburgh, Pennsylvania, United States of America; 2 Constructive Dialogue Institute, New York, New York, United States of America; 3 Teachers College, Columbia University, New York, New York, United States of America; Hiroshima International University: Hiroshima Kokusai Daigaku, JAPAN

## Abstract

Conflict is a ubiquitous, but potentially destructive, feature of social life. In the current research, we argue that intellectual humility—the awareness of one’s intellectual fallibility—plays an important role in promoting constructive responses and decreasing destructive responses to conflict in different contexts. In Study 1, we examine the role of intellectual humility in interpersonal conflicts with friends and family members. In Study 2, we extend this finding to workplace conflicts. In both studies we find that intellectual humility predicts more constructive and less destructive responses to conflict. This work extends the burgeoning literature on the benefits of intellectual humility by demonstrating its association with responses that help defuse conflictual encounters.

## Introduction

*“In the course of my life, I have often had to eat my words, and I must confess that I have always found it a wholesome diet.”*

*–Winston Churchill*

Whether it’s because of a misunderstanding, a betrayal, or an insult, we all frequently face conflict situations that have the capacity to fracture our personal and professional relationships. How we choose to engage with the other person during these conflicts determines whether they escalate into destructive events or de-escalate and potentially become opportunities for learning and relationship growth. In the current research, we examine how one individual difference factor, *intellectual humility*, predicts constructive responses to interpersonal conflict in different relationship contexts.

### The outcomes of destructive conflict

Interpersonal conflict can be costly when managed ineffectively. When occurring with our close relationship partners, conflict is often experienced as physically and psychologically stressful. For example, persistent marital conflict is associated with chronic health issues (e.g., high blood pressure) and reduced immune functioning [1]. Conflict and hostility can also lead to divorce and separation [2]. When married couples have children, their negative conflict patterns or the dissolution of their relationship can disrupt their children’s academic, psychological, physical, and social wellbeing [3–5]. Beyond romantic relationships, conflict is also one of the strongest predictors of friendship dissolution [6].

Interpersonal workplace conflict can also have deleterious consequences. Conflict with coworkers is associated with lower workplace satisfaction and organizational commitment, as well as higher intention to turnover [7], costing organizations millions of dollars annually [e.g., 8]. Workplace conflict can also spiral into more extreme incivility [9] and acts of revenge [10]. In the most extreme cases, workplace conflict can even escalate into outright aggression [11]. A recent poll found that even in remote work environments, 80% of respondents had experienced workplace conflict, including 67% who reported being aggressively cursed at by a colleague [12].

Thus, across different relationship contexts, conflict can have powerful destructive outcomes. It is therefore paramount to understand factors that support constructive responses aimed at defusing these conflicts and promoting more harmonious social functioning.

### The benefits of constructive conflict

Despite the challenges and costs associated with conflict, these encounters need not be destructive [13]. When people engage in collaboration, problem-solving, and open-minded, non-hostile communication, conflict can be less damaging and can even have productive outcomes [14–16]. For example, actively collaborating to resolve relationship conflict is associated with positive feelings between relationship partners as well as short- and long-term benefits to the relationship [17]. In the workplace, minority dissent in teams can lead to more innovation [18], especially when that disagreement is paired with openness and a safe climate [19]. This raises the question: how can we minimize the costs of destructive conflict and encourage more constructive cognitions and behaviors? We propose that intellectual humility is one solution.

### Intellectual humility in conflict

Intellectual humility (IH) has been growing as an area of research over the last decade [20]. While researchers vary in how they define IH, most agree that a central feature is an awareness of one’s intellectual fallibility [20–22]. IH is generally considered a trait [20], though it can also vary across situations [23].

Research provides emerging support for the possibility that IH plays an important role in driving constructive conflict management. During interpersonal conflict, people tend to adopt a narrow perspective that focuses on their own experience rather than the other person’s experience [24, 25]. This limited perspective often leads to misattributions, blaming, and conflict escalation [24]. Because people with high IH acknowledge that their viewpoint is limited and potentially flawed, they tend to be motivated to seek out other perspectives and to favor a more nuanced view of the conflict at hand [26–28]. In support of this argument, people with higher IH are dispositionally more openminded [29], are more likely to empathize with others during a disagreement [30], and are more likely to offer comprehensive apologies when they have harmed someone, at least in part because they feel more empathy for the victim [31].

Despite the growth in literature on IH and correlates of constructive conflict behavior (e.g., empathy, apologies, etc.), little work has examined if IH predicts how we behave and think in conflict. The existing work on this mostly comes from research on wise reasoning—a construct containing IH, appreciation for contexts, sensitivity to changes in the relationship, and searching for compromise [32–34]. Researchers in this area have found that wise reasoning predicts cooperation in economic game tasks [35] and feelings of positivity about interpersonal conflicts [36]. According to this framework, IH might be associated with more constructive conflict responses (e.g., compromise) because they are part of the same overarching construct of wise reasoning [35]. In the current research we hope to replicate and extend these findings by examining if IH predicts specific strategies during conflicts in different domains.

### The current research

The potential for IH to promote more constructive responses to conflict is an exciting advance that may point to a fulcrum for future intervention. In the current research, we replicate and extend previous findings on IH in the domain of interpersonal conflict to provide more robust evidence for this possibility. To do this, we examine data from Perspectives, a program developed by the Constructive Dialogue Institute—a nonprofit organization that offers online training programs to reduce ideological intolerance. Both samples include data from pre-surveys completed prior to the content of the Perspectives program. Data was derived from a larger set of studies, portions of which are reported by Welker and colleagues [37] in a paper examining whether the Perspectives program causes improvements in IH, affective polarization, and conflict resolution. After participants signed up, they were taken to a short online survey containing demographic items and measures of outcomes the Perspectives program was expected to impact. Although Welker and colleagues briefly reported simple bivariate correlations between the General IH scale and the conflict responses subscales for both studies reported here, we expand on this finding in this paper because 1) examining this relationship in depth was not the goal of the Welker et al. paper and several aspects of this relationship were not explored in that paper, analytically or theoretically, 2) it is critical to understand the behavioral correlates of IH in depth and the goal of this paper is to unpack that relationship, and 3) we are conducting more sophisticated analyses, such as multilevel modeling with random intercepts, and this will lead to a better understanding of the relationship between IH and conflict resolution than previously offered.

Our first study tested the role of IH in conflicts with friends and family members. We replicated and also extended this in Study 2 with a workplace sample to test the role of IH in workplace conflicts. In these studies, participants thought of specific people with whom they have conflict and reported on their behaviors and emotions in the context of these disagreements. Across both studies we find support for IH as a predictor of constructive conflict responses.

In this research, we used two different IH scales. The first is the General Intellectual Humility Scale (GIHS; [22]), which is a unidimensional scale focused on seeing one’s views and beliefs as fallible. It therefore only assesses one’s internal recognition of fallibility. The second is the Comprehensive Intellectual Humility Scale (CIHS; [38]), which is a multidimensional scale that includes four subscales: independence of intellect and ego, openness to revising one’s views, respect for others’ viewpoints, and lack of intellectual overconfidence. CIHS therefore assesses both internal aspects of IH, as well as more relational and other-focused manifestations of IH. While the GIHS and CIHS are typically correlated with each other, they are thought to capture different aspects and conceptualizations of IH. We therefore included both scales to provide more evidence and nuance to the correlations of IH in this domain. It is common to keep these scales separate when including both in a study [e.g., 39]. We did not have *a priori* expectations about how these scales might differ, but did test for differences between each scale’s predictive ability.

We hypothesized that IH would be associated with more constructive and less destructive conflict styles in all contexts. Because these are secondary data analyses, there were several measures assessed in each study that were unrelated to the current research question or measured in only part of the study sample. For concision, we report only the main variables of interest below. These studies were not preregistered. Full materials, data, and code for all studies are available at https://osf.io/sjh97/?view_only=1ec9acad7dd043fe8a5a7f0324aad5ab.

## Study 1

In Study 1, we tested the role of IH in conflicts with friends and family members. We hypothesized that IH would be associated with more constructive and less destructive conflict strategies.

### Method

#### Participants

In Study 1, we analyzed the pre-survey data from a Perspectives higher education randomized control trial. Data collection occurred between August 23, 2021 and May 2, 2022. Participants were recruited from ten classes within three higher education institutions (one large Southern university, one large Eastern university, and one small Western community college). All participants completed an online consent form and this study was approved by the IRBs at University of North Texas, Crafton Hills College, and the University of Maryland. The total sample included 775 participants. We removed those who did not complete the survey (*n* = 66), and then those who failed the attention check (*n* = 69). This left a final sample of 640 participants (*M*_*age*_ = 21.05, *SD*_*age*_ = 3.54; Female = 326, Male = 135, non-binary = 17, chose to self-describe = 3, did not report = 159; African American/Black = 58, East or Southeast Asian = 35, Hispanic/Latino = 76, Middle Eastern/North African = 4, South Asian = 17, White/Caucasian = 226, indicated more than one racial identity = 61, Other = 1, Prefer not to say = 2, did not report = 160). A sensitivity analysis in G Power [40] revealed that the study was powered to detect small-medium correlations (*ρ* = .14, 95% power, *α* = .05).

#### Materials and procedure

*Intellectual humility*. Participants completed the six-item GIHS (e.g., “I accept that my beliefs and attitudes may be wrong”) on a scale from 1 (*Not at all like me*) to 5 (*Very much like me*; *α* = .85). Participants also completed the 22-item CIHS (e.g., I’m willing to change my mind once it’s made up about an important topic”) on a scale from 1 (*Strongly disagree*) to 5 (*Strongly agree*; *α* = .81).

*Conflict responses*. Participants then completed items assessing their behaviors during conflict [41]. Participants thought of a friend or family member with whom they have conflict. They then reported on the behavior and communication skills they usually employ, divided into whether these behaviors and constructive/positive or destructive/negative. They completed items assessing subscales of positive informing (*α* = .61; three items, e.g., “When in conflict with PERSON, I openly discuss what is important to me so that others can understand me.”), positive evading (*α* = .57; three items, e.g., “When in conflict with PERSON, I suggest that a problem be discussed at a later time to give people more time to consider various alternatives.”), positive opening (*α* = .85; six items, e.g., “When in conflict with PERSON, I try to find out about what is most important on the other side before suggesting possible solutions.”), positive uniting (*α* = .87; seven items, e.g., “When in conflict with PERSON, I when possible, treat the problem as one that can be solved by working together.”), negative attacking (*α* = .84; eight items, e.g., “When in conflict with PERSON, I speak in a disrespectful manner.”) and negative evading (*α* = .69; four items, e.g., “When in conflict with PERSON, I remain silent or change the subject because I am uncomfortable with open conflict.”) on a scale from 1 (*Never*) to 7 (*Always*). Because of a substantial amount of overlap between subscales, we created overall composites of positive behaviors (*α* = .91) and negative behaviors (*α* = .77) as our primary outcomes (see Table 1 for correlations across subscales).

**Table 1:** Correlations between subscales, Study 1.

	1	2	3	4	5	6	7	8
1. GIHS	—							
2. CIHS	.58***	—						
3. Positive Inform	.21***	.13**	—					
4. Positive Evade	.20***	.13**	.16***	—				
5. Positive Open	.34***	.34***	.52***	.35***	—			
6. Positive Unite	.30***	.31***	.51***	.31***	.85***	—		
7. Negative Attack	-.17***	-.28***	.01	-.04	-.34***	-.29***	—	
8. Negative Evade	.05	-.04	-.16***	.43***	-.03	-.06	.12*	—
*Mean*	3.92	3.72	5.01	4.04	4.84	4.75	3.11	3.86
*SD*	.63	.39	1.05	1.20	1.06	1.11	1.04	1.20

*Brief socially desirability scale*. To ensure that the associations in this study were not due to socially desirable responding, participants also completed a five-item measure of social desirability [42]. This measure asks participants questions with an unlikely, but socially desirable response (e.g., “Do you always practice what you preach?") Participants answered each question with answer options of yes, no, or prefer not to say. We coded socially desirable answers as 1, socially undesirable answers as 0, and excluded participants who indicated they would prefer not to respond. We then averaged across the five items to create a composite score (*α* = .44).

### Data analyses

To account for data clustering, we ran multi-level models using lme4 [43] and lmerTest [44] in R version 4.2.2 [45]. We first included random intercepts for both higher education institution (*N*_institution_ = 3) and class (*N*_class_ = 10), however this often resulted in singular fit. Because even small ICCs can lead to biased results [46], we included random intercepts for class whenever the ICC was above 0 (see S2 Table in S1 File).

To test for differences between the GIHS and CIHS, we computed fisher *z* scores [47].

### Results

We regressed positive and negative conflict responses on each IH scale (see Table 2). Both GIHS and CIHS predicted more positive behaviors and fewer negative behaviors. The effect sizes for both scales ranged from small to medium [48]. We retested both models controlling for age and gender. The association between GIHS and the negative composite fell to just below significance (*p* = .057; see SM).

**Table 2:** Regression models using intellectual humility as a predictor, Study 1.

	*Positive Composite*									
	*β*	*b*	*95% CI*	*SE*	*p*	*β*	*b*	*95% CI*	*SE*	*p*
(Intercept)	0.06	2.75	2.23, 3.27	0.26	< .001	0.04	2.13	1.39, 2.87	0.38	< .001
GIHS	0.36	0.51	0.39, 0.64	0.06	< .001					
CIHS						0.32	0.70	0.51, 0.90	0.10	< .001
σ^2^	0.69					0.71				
τ_00 Class_	0.03					0.02				
ICC	0.04					0.02				
N_Class_	10					10				
Observations	426					426				
Marginal R^2^	.127					.104				
	*Negative Composite*									
	*β*	*b*	*95% CI*	*SE*	*p*	*β*	*b*	*95% CI*	*SE*	*p*
(Intercept)	0.000	3.96	3.46, 4.46	0.26	< .001	0.000	5.22	4.52, 5.93	0.36	< .001
GIHS	-0.11	-0.15	-0.28, -0.03	0.06	.018					
CIHS						-0.25	-0.50	-0.69, -0.31	0.10	< .001
Observations	426					426				
Marginal R^2^	.013					.061				

Fisher *z* scores revealed that CIHS had a significantly stronger correlation with the negative composite than did GIHS, *z* = 3.09, *p* = .002. The associations with the positive composite did not differ between scales.

Finally, to ensure that these results were not due to socially desirable responding, we retested both models controlling for the brief social desirability scale. Again, the association between GIHS and the negative composite fell to just below significance (*p* = .065; see SM for full results).

### Discussion

Both IH scales predicted more productive conflict behaviors and strategies. Interestingly, CIHS appeared to be a better predictor of the negative composite than the GIHS. This may indicate that the GIHS and CIHS predict positive responses to interpersonal conflicts in similar ways, but that CIHS maps on more strongly to negative conflict responses. We tested whether this pattern replicated in Study 2.

## Study 2

In Study 1, we showed benefits of IH for more constructive responding to interpersonal conflicts with family and friends. In Study 2, we aimed to replicate the effects of Study 1, while also extending into the domain of workplace conflict. To do so, we examined the relationship between IH and constructive responses to workplace conflict among members of government finance organizations. Study 2 used a pre-survey from a second randomized control trial with a new sample.

### Method

#### Participants

The total sample included 277 participants who were all members of the Government Finance Officers Organization and worked for local governments in the United States. Data collection occurred between August 16, 2021 and October 18, 2021. This study was determined as exempt from requiring consent by Sterling IRB. Participants were provided with information prior to participating, including an overview of the study procedure and time commitment, the privacy policy, and contact information if they had any questions. We used this sample from the workforce because it allowed us to focus on conflict between coworkers. We removed those who did not complete the survey (*n* = 8). This left a final sample of 269 participants (*M*_*age*_ = 49.79, *SD*_*age*_ = 9.62; Female = 190, Male = 69, “prefer not to say” = 2, did not report = 8; African American/Black = 9, East or Southeast Asian = 7, Hispanic/Latino = 10, South Asian = 1, White/Caucasian = 198, indicated more than one racial identity = 7, Other = 2, “Prefer not to say” = 4, did not report = 31). Most participants identified as executives or department heads (*n* = 138), with others identifying as middle managers (*n* = 57), staff (*n* = 36), or elected officials (*n* = 7; failed to report = 31). A sensitivity analysis revealed that the study was powered to detect small-medium correlations (*ρ*_*full sample*_ = .22, 95% power, *α* = .05).

*Materials and procedure*. Participants completed the same measures as in Study 1, including full versions of the GIHS (*α* = .83) and CIHS (*α* = .84), and conflict responses while thinking about conflicts with a family member or friend (*α*_*positive composite*_ = .91; *α*_*negative composite*_ = .84; see Table 3 for correlations across subscales). In addition, they completed conflict responses while thinking about conflicts with a work supervisor and supervisee (*α*_*positive composite*_ = .95; *α*_*negative composite*_ = .90).

**Table 3:** Correlations between primary variables, Study 2.

	1	2	3	4	5	6	7	8	9	10	11	12	13	14
1. GIHS	—													
2. CIHS	.54***	—												
3. Positive Inform (Family)	.31***	.24**	—											
4. Positive Evade (Family)	.18*	.04	.25**	—										
5. Positive Open (Family)	.34***	.31***	.49***	.38***	—									
6. Positive Unite (Family)	.34***	.28***	.60***	.47***	.82***	—								
7. Negative Attack (Family)	-.17*	-.25***	-.21**	-.20**	-.63***	-.63***	—							
8. Negative Evade (Family)	-.16*	-.23**	-.40***	.24**	-.21**	-.24**	.21**	—						
9. Positive Inform (Work)	.27***	.30***	.51***	.18*	.39***	.33***	-.11	-.26***	—					
10. Positive Evade (Work)	.07	.05	.16*	.48***	.22**	.22**	-.08	.23**	.20**	—				
11. Positive Open (Work)	.28***	.25**	.38***	.26**	.62***	.47***	-.28***	-.14	.63***	.26***	—			
12. Positive Unite (Work)	.37***	.28***	.46***	.25**	.52***	.50***	-.24**	-.14	.68***	.26***	.85***	—		
13. Negative Attack (Work)	-.13	-.36***	-.05	.06	-.33***	-.24**	.45***	.18*	-.08	.02	-.49***	-.37***	—	
14. Negative Evade (Work)	-.10	-.26**	-.15	.25**	-.17*	-.09	.25**	.53***	-.32***	.33***	-.22**	-.18*	.34***	—
*Mean*	3.57	3.84	4.98	4.32	4.59	4.96	3.18	4.09	5.17	4.51	5.29	5.54	2.03	3.44
*SD*	0.62	0.33	1.01	0.95	0.97	0.89	1.09	1.21	0.82	0.89	0.82	0.71	0.76	1.09

### Data analyses

To account for data clustering, we included random intercepts for department area whenever the ICC was above 0 (see S8 Table in S1 File).

### Results

We regressed each family/friend conflict composite on each IH scale (see Table 4). Replicating Study 1, both GIHS and CIHS predicted more positive behaviors and fewer negative behaviors in the friend/family conflicts. We then regressed workplace conflict behaviors on each IH scale. Both GIHS and CIHS predicted more positive behaviors in the workplace conflict. Although GIHS was associated in the expected direction, only CIHS significantly predicted fewer negative behaviors. We retested all models controlling for age and gender. All significant associations remained significant.

**Table 4:** Regression models using intellectual humility as a predictor, Study 2.

	*Positive Composite (Family/Friends)*									
	*β*	*b*	*95% CI*	*SE*	*p*	*β*	*b*	*95% CI*	*SE*	*p*
(Intercept)	-0.00	3.06	2.42, 3.70	.32	< .001	-0.00	1.96	0.60, 3.33	.69	.005
GIHS	0.37	0.47	0.30, 0.65	.09	< .001					
CIHS						0.29	0.73	0.37, 1.08	.18	< .001
Observations	173					173				
R^2^	.140					.087				
	*Negative Composite (Family/Friends)*									
	*β*	*b*	*95% CI*	*SE*	*p*	*β*	*b*	*95% CI*	*SE*	*p*
(Intercept)	-0.00	4.56	3.78, 5.33	.39	< .001	-0.00	6.78	5.22, 8.35	.79	< .001
GIHS	-0.21	-0.30	-0.52, -0.09	.11	.006					
CIHS						-0.30	-0.86	-1.27, -0.46	.21	< .001
Observations	173					173				
R^2^	.043					.093				
	*Positive Composite (Work)*									
	*β*	*b*	*95% CI*	*SE*	*p*	*β*	*b*	*95% CI*	*SE*	*p*
(Intercept)	-0.14	3.88	3.28, 4.48	.31	< .001	-0.12	3.06	1.91, 4.21	.58	< .001
GIHS	0.33	0.35	0.20, 0.51	.08	< .001					
CIHS						0.27	0.54	0.25, 0.84	.15	< .001
σ^2^	0.35					0.36				
τ_00 AreaDept_	0.04					0.03				
ICC	0.10					0.09				
N _AreaDept_	5					5				
Observations	160					160				
Marginal R^2^	.102					.072				
	*Negative Composite (Work)*									
	*β*	*b*	*95% CI*	*SE*	*p*	*β*	*b*	*95% CI*	*SE*	*p*
(Intercept)	0.01	3.11	2.43, 3.80	.35	< .001	0.00	5.79	4.54, 7.04	.63	< .001
GIHS	-0.14	-0.17	-0.36, 0.02	.10	.075					
CIHS						-0.38	-0.86	-1.18, -0.53	.16	< .001
σ^2^	0.51					0.44				
τ_00 AreaDept_	0.00					0.00				
ICC	0.01					0.00				
N _AreaDept_	5					5				
Observations	160					160				
Marginal R^2^	.020					.147				

Fisher *z* scores revealed that CIHS had a significantly stronger correlation with the workplace negative composite (*z* = 3.49, *p* < .001) than did GIHS. All other correlations were statistically similar.

### Discussion

Both IH scales predicted more constructive and less destructive behaviors during conflicts with family, friends, and coworkers. GIHS was significantly associated with more positive behaviors in both contexts, and fewer negative behaviors in conflicts with family and friends. CIHS was significantly associated with more positive behaviors and fewer negative behaviors in both contexts. Combined with Study 1, these results suggest that IH is an important predictor of conflict responses in conflicts with family, friends, and coworkers, but suggest that the CIHS might be a more reliable predictor of negative conflict behaviors than the GIHS.

## General discussion

Conflicts are a normal and common part of life. Left unresolved, however, even small conflicts can escalate and have harmful consequences for the parties involved. It is therefore critical that we understand how to promote more constructive responses to the conflicts we typically encounter, such as interpersonal and workplace disagreements. Across two studies, we found that people with high IH were more likely to engage in constructive conflict strategies and less likely to engage in destructive conflict strategies. We found support for associations with IH across conflicts with family and friends (Studies 1 and 2) and workplace colleagues (Study 2).

Although the current research replicates and extends the existing work in important ways, it has several limitations. First, all our findings were self-reported. Although self-reports and hypothetical scenarios are both limited by their reflected rather than behavioral nature, prior work on IH shows similar patterns of associations when using both self-reported vignette and behavioral paradigms [e.g., 49, 50]. We therefore have confidence that the self-reported tendencies of high IH people mostly translate to real behavior. Nevertheless, future research should investigate whether IH predicts real behavior during conflict interactions. Second, all our findings are limited by their correlational nature. While most research on IH relies on correlational evidence, future work might leverage newly developed manipulations that temporarily boost IH [51, 52] to conduct experimental replications. Third, our samples and results can only speak to the United States context and people participating through the Constructive Dialogue Institute programming. It is therefore possible that some participants selected into the Constructive Dialogue Institute Programming precisely because they feel strongly about discussing across differences. This seems unlikely to have made a difference in the results, however, because a large proportion of the academic samples participated in the programming as part of their classes as opposed to participating of their own interest. Nevertheless, future work should seek to replicate these results in other countries and with other samples.

Despite these limitations, the current research finds evidence for the association between IH and conflict responses across different conflict contexts. Future work might examine whether intervening at the level of people’s IH promotes enduring improvements to how people engage with their conflict partners. Future work could also build on this by examining the impacts of *perceived* IH during conflict. In a practical sense, perceiving IH in another party might signal that they are willing to listen and collaborate. This might encourage collaborative behaviors and IH from the perceiver. In line with this possibility, perceptions of conversational receptiveness—a construct theoretically similar to IH—increases collaboration [53] and reciprocal levels of receptiveness in the listener [54]. IH may prove to be similarly contagious during conflicts. Finally, future research might investigate if and when IH could backfire during conflict. For example, could someone with high IH see a low IH counterpart as unworthy of collaboration [e.g., 55]? Could someone with high IH be seen as deferential and be taken advantage of during conflict? In a time of polarization and intense ideological and personal conflict, it is important to understand when IH is beneficial and when it might not be.

## Supporting information

S1 File IH and conflict SM, supplementary analyses and tables, Studies 1 and 2. (DOCX)
