# Supplementary material for: Intellectual humility is reliably associated with constructive responses to conflict
Source: PLoS One. 2024 Sep 6;19(9):e0309848. doi: 10.1371/journal.pone.0309848 (PMC11379209; doi:10.1371/journal.pone.0309848)
Supplement: S1 File — (DOCX) [file pone.0309848.s001.docx]

**Supplementary Materials**

Additional Analyses: Study 1………………….……………..…………………………...........p. 2

Additional Analyses: Study 2……………….……..………..……………………….…..........p. 11

**Additional Analyses: Study 1**

**S1 Table. Regression models using intellectual humility as a predictor on all conflict subscales, Study 1**

|  | *Positive Informing* | | | | | | | | | |
| --- | --- | --- | --- | --- | --- | --- | --- | --- | --- | --- |
|  | *β* | *b* | *95% CI* | *SE* | *p* | *β* | *b* | *95% CI* | *SE* | *p* |
| (Intercept) | 0.00 | 3.65 | 3.03, 4.26 | 0.31 | <.001 | 0.00 | 3.78 | 2.89, 4.68 | 0.45 | <.001 |
| GIHS | 0.21 | 0.35 | 0.19, 0.50 | 0.08 | <.001 |  |  |  |  |  |
| CIHS |  |  |  |  |  | 0.13 | 0.33 | 0.09, 0.57 | 0.12 | .007 |
|  |  |  |  |  |  |  |  |  |  |  |
| Observations | 425 |  |  |  |  | 425 |  |  |  |  |
| R^2^ | .044 |  |  |  |  | .017 |  |  |  |  |
|  | *Positive Evading* | | | | | | | | | |
|  | *β* | *b* | *95% CI* | *SE* | *p* | *β* | *b* | *95% CI* | *SE* | *p* |
| (Intercept) | -0.00 | 2.53 | 1.82, 3.23 | 0.36 | <.001 | -0.00 | 2.62 | 1.59, 3.64 | 0.52 | <.001 |
| GIHS | 0.20 | 0.38 | 0.21, 0.56 | 0.09 | <.001 |  |  |  |  |  |
| CIHS |  |  |  |  |  | 0.13 | 0.38 | 0.11, 0.65 | 0.14 | .006 |
|  |  |  |  |  |  |  |  |  |  |  |
| Observations | 425 |  |  |  |  | 425 |  |  |  |  |
| R^2^ | .041 |  |  |  |  | .017 |  |  |  |  |
|  | *Positive Opening* | | | | | | | | | |
|  | *β* | *b* | *95% CI* | *SE* | *p* | *β* | *b* | *95% CI* | *SE* | *p* |
| (Intercept) | 0.04 | 2.58 | 1.97, 3.18 | 0.31 | <.001 | 0.01 | 1.59 | 0.73, 2.45 | 0.44 | <.001 |
| GIHS | 0.35 | 0.58 | 0.43, 0.73 | 0.08 | <.001 |  |  |  |  |  |
| CIHS |  |  |  |  |  | 0.34 | 0.88 | 0.65, 1.11 | 0.12 | <.001 |
|  |  |  |  |  |  |  |  |  |  |  |
| σ^2^ | 0.98 |  |  |  |  | 0.99 |  |  |  |  |
| τ_00_ _Class_ | 0.02 |  |  |  |  | 0.01 |  |  |  |  |
| ICC | 0.02 |  |  |  |  | 0.01 |  |  |  |  |
| N_Class_ | 10 |  |  |  |  | 10 |  |  |  |  |
| Observations | 426 |  |  |  |  | 426 |  |  |  |  |
| Marginal R^2^ | .120 |  |  |  |  | .117 |  |  |  |  |
|  | *Positive Uniting* | | | | | | | | | |
|  | *β* | *b* | *95% CI* | *SE* | *p* | *β* | *b* | *95% CI* | *SE* | *p* |
| (Intercept) | 0.08 | 2.62 | 1.95, 3.28 | 0.34 | <.001 | 0.05 | 1.66 | 0.75, 2.59 | 0.47 | <.001 |
| GIHS | 0.32 | 0.56 | 0.41, 0.72 | 0.08 | <.001 |  |  |  |  |  |
| CIHS |  |  |  |  |  | 0.32 | 0.85 | 0.61, 1.09 | 0.12 | <.001 |
|  |  |  |  |  |  |  |  |  |  |  |
| σ^2^ | 1.07 |  |  |  |  | 1.08 |  |  |  |  |
| τ_00_ _Class_ | 0.08 |  |  |  |  | 0.06 |  |  |  |  |
| ICC | 0.07 |  |  |  |  | 0.05 |  |  |  |  |
| N_Class_ | 10 |  |  |  |  | 10 |  |  |  |  |
| Observations | 426 |  |  |  |  | 426 |  |  |  |  |
| Marginal R^2^ | .099 |  |  |  |  | .098 |  |  |  |  |
|  | *Negative Attacking* | | | | | | | | | |
|  | *β* | *b* | *95% CI* | *SE* | *p* | *β* | *b* | *95% CI* | *SE* | *p* |
| (Intercept) | -0.00 | 4.22 | 3.60, 4.83 | 0.31 | <.001 | -0.00 | 5.73 | 4.87, 6.59 | 0.44 | <.001 |
| GIHS | -0.17 | -0.28 | -0.44, -0.13 | 0.08 | <.001 |  |  |  |  |  |
| CIHS |  |  |  |  |  | -0.28 | -0.70 | -0.93, -0.47 | 0.12 | <.001 |
|  |  |  |  |  |  |  |  |  |  |  |
| Observations | 426 |  |  |  |  | 426 |  |  |  |  |
| R^2^ | .030 |  |  |  |  | .079 |  |  |  |  |
|  | *Negative Evading* | | | | | | | | | |
|  | *β* | *b* | *95% CI* | *SE* | *p* | *β* | *b* | *95% CI* | *SE* | *p* |
| (Intercept) | -0.00 | 3.49 | 2.76, 4.21 | 0.37 | <.001 | -0.01 | 4.29 | 3.25, 5.33 | 0.53 | <.001 |
| GIHS | 0.05 | 0.10 | -0.09, 0.28 | 0.09 | .302 |  |  |  |  |  |
| CIHS |  |  |  |  |  | -0.04 | -0.12 | -0.39, 0.16 | 0.14 | .406 |
|  |  |  |  |  |  |  |  |  |  |  |
| σ^2^ | 1.45 |  |  |  |  | 1.45 |  |  |  |  |
| τ_00_ _Class_ | 0.00 |  |  |  |  | 0.01 |  |  |  |  |
| ICC | 0.00 |  |  |  |  | 0.00 |  |  |  |  |
| N_Class_ | 10 |  |  |  |  | 10 |  |  |  |  |
| Observations | 426 |  |  |  |  | 426 |  |  |  |  |
| Marginal R^2^ | .003 |  |  |  |  | .002 |  |  |  |  |

**S2 Table. ICCs for empty models, Study 1**

| *Outcome* | *Class ICC* |
| --- | --- |
| Positive Inform | .000 |
| Positive Evade | .000 |
| Positive Open | .003 |
| Positive Unite | .043 |
| Positive Composite | .017 |
| Negative Attack | .000 |
| Negative Evading | .003 |
| Negative Composite | .000 |

**S3 Table. Model Fit Statistics, Study 1**

| Outcome | Model Type | AIC | BIC | Log Likelihood | χ^2^ test against empty model (df in parentheses) |
| --- | --- | --- | --- | --- | --- |
| Positive Open | Empty Model | 1263.5 | 1275.7 | -628.78 |  |
|  | Including GIHS | 1211.8 | 1228.0 | -601.90 | 53.75 (1) ^***^ |
| Positive Open | Empty Model | 1263.5 | 1275.7 | -628.78 |  |
|  | Including CIHS | 1212.6 | 1228.8 | -602.29 | 52.97 (1) ^***^ |
| Positive Unite | Empty Model | 1299.8 | 1311.9 | -646.89 |  |
|  | Including GIHS | 1255.6 | 1271.8 | -623.79 | 46.19 (1) ^***^ |
| Positive Unite | Empty Model | 1299.8 | 1311.9 | -646.89 |  |
|  | Including CIHS | 1256.2 | 1272.5 | -624.12 | 45.54 (1) ^***^ |
| Positive Composite | Empty Model | 1122.4 | 1134.5 | -558.19 |  |
|  | Including GIHS | 1066.3 | 1082.5 | -529.16 | 58.07 (1) ^***^ |
| Positive Composite | Empty Model | 1122.4 | 1134.5 | -558.19 |  |
|  | Including CIHS | 1077.0 | 1093.2 | -534.50 | 47.39 (1) ^***^ |
| Negative Evading | Empty Model | 1371.8 | 1384.0 | -682.91 |  |
|  | Including GIHS | 1372.7 | 1388.9 | -682.36 | 1.11 (1) |
| Negative Evading | Empty Model | 1371.8 | 1384.0 | -682.91 |  |
|  | Including CIHS | 1373.2 | 1389.4 | -682.59 | 0.66 (1) |

**Conflict Emotions**

Participants also completed eight items assessing negative emotions during conflict (e.g., “In general, when I am engaged in conflict with PERSON, I feel frustrated / irritated,” α = .87) and four items assessing positive emotions during conflict (e.g., “In general, when I am engaged in conflict with PERSON, I feel hopeful,” *α* = .60) on a scale from 1 (*Never*) to 7 (*Always;* Coleman & Lim, 2001). Neither CIHS nor GIHS predicted more positive emotion. CIHS, but not GIHS predicted less negative emotion during conflict (see S4 Table).

**S4 Table. Regression models using intellectual humility as a predictor of conflict emotions, Study 1**

|  | *Positive Conflict Emotion* | | | | | | | | | |
| --- | --- | --- | --- | --- | --- | --- | --- | --- | --- | --- |
|  | *β* | *b* | *CI* | *SE* | *p* | *β* | *b* | *CI* | *SE* | *p* |
| (Intercept) | .18 | 3.51 | 2.83, 4.20 | .35 | <.001 | .18 | 3.76 | 2.82, 4.70 | .48 | <.001 |
| GIHS | .06 | .10 | -0.05, 0.26 | .08 | .196 |  |  |  |  |  |
| CIHS |  |  |  |  |  | .02 | .04 | -0.20, 0.28 | .12 | .733 |
|  |  |  |  |  |  |  |  |  |  |  |
| σ^2^ | 1.01 |  |  |  |  | 1.01 |  |  |  |  |
| τ_00_ _Class_ | 0.16 |  |  |  |  | 0.15 |  |  |  |  |
| ICC | 0.14 |  |  |  |  | 0.13 |  |  |  |  |
| N_Class_ | 10 |  |  |  |  | 10 |  |  |  |  |
| Observations | 412 |  |  |  |  | 412 |  |  |  |  |
| Marginal R^2^ | .004 |  |  |  |  | .000 |  |  |  |  |
|  | *Negative Conflict Emotion* | | | | | | | | | |
|  | *β* | *b* | *CI* | *SE* | *p* | *β* | *b* | *CI* | *SE* | *p* |
| (Intercept) | -.04 | 4.22 | 3.54, 4.89 | .34 | <.001 | -.03 | 5.25 | 4.29, 6.20 | .48 | <.001 |
| GIHS | -.05 | -.09 | -0.26, 0.07 | .08 | .270 |  |  |  |  |  |
| CIHS |  |  |  |  |  | -.14 | -.37 | -0.62, -0.12 | .13 | .004 |
|  |  |  |  |  |  |  |  |  |  |  |
| σ^2^ | 1.18 |  |  |  |  | 1.16 |  |  |  |  |
| τ_00_ _Class_ | 0.03 |  |  |  |  | 0.03 |  |  |  |  |
| ICC | 0.03 |  |  |  |  | 0.03 |  |  |  |  |
| N_Class_ | 10 |  |  |  |  | 10 |  |  |  |  |
| Observations | 418 |  |  |  |  | 418 |  |  |  |  |
| Marginal R^2^ | .003 |  |  |  |  | .020 |  |  |  |  |

**Analyses Controlling for Brief Social Desirability Scale (BSDS)**

**S5 Table. Regression models using intellectual humility as a predictor and controlling for social desirability, Study 1**

|  | *Positive Informing* | | | | | | | | | |
| --- | --- | --- | --- | --- | --- | --- | --- | --- | --- | --- |
|  | *β* | *b* | *95% CI* | *SE* | *p* | *β* | *b* | *95% CI* | *SE* | *p* |
| (Intercept) | 0.00 | 3.54 | 2.87, 4.22 | 0.34 | <.001 | -0.00 | 3.81 | 2.83, 4.79 | 0.50 | <.001 |
| BSDS | 0.07 | 0.34 | -0.11, 0.79 | 0.23 | .141 | 0.09 | 0.39 | -0.07, 0.85 | 0.23 | .098 |
| GIHS | 0.20 | 0.33 | 0.17, 0.50 | 0.08 | <.001 |  |  |  |  |  |
| CIHS |  |  |  |  |  | 0.11 | 0.27 | 0.01, 0.53 | 0.13 | .038 |
|  |  |  |  |  |  |  |  |  |  |  |
| Observations | 377 |  |  |  |  | 377 |  |  |  |  |
| R^2^ | .048 |  |  |  |  | .020 |  |  |  |  |
|  | *Positive Evading* | | | | | | | | | |
|  | *β* | *b* | *95% CI* | *SE* | *p* | *β* | *b* | *95% CI* | *SE* | *p* |
| (Intercept) | -0.00 | 2.42 | 1.64, 3.20 | 0.40 | <.001 | -0.00 | 2.45 | 1.31, 3.59 | 0.58 | <.001 |
| BSDS | -0.05 | -0.28 | -0.81, 0.24 | 0.27 | .290 | -0.04 | -0.23 | -0.77, 0.30 | 0.27 | .390 |
| GIHS | 0.22 | 0.43 | 0.24, 0.62 | 0.10 | <.001 |  |  |  |  |  |
| CIHS |  |  |  |  |  | 0.15 | 0.44 | 0.14, 0.74 | 0.15 | .004 |
|  |  |  |  |  |  |  |  |  |  |  |
| Observations | 377 |  |  |  |  | 377 |  |  |  |  |
| R^2^ | .051 |  |  |  |  | .023 |  |  |  |  |
|  | *Positive Opening* | | | | | | | | | |
|  | *β* | *b* | *95% CI* | *SE* | *p* | *β* | *b* | *95% CI* | *SE* | *p* |
| (Intercept) | 0.06 | 2.39 | 1.73, 3.04 | 0.33 | <.001 | 0.00 | 1.45 | 0.53, 2.38 | 0.47 | .002 |
| BSDS | 0.12 | 0.57 | 0.13, 1.00 | 0.22 | .010 | 0.13 | 0.60 | 0.17, 1.04 | 0.22 | .007 |
| GIHS | 0.34 | 0.57 | 0.41, 0.73 | 0.08 | <.001 |  |  |  |  |  |
| CIHS |  |  |  |  |  | 0.32 | 0.83 | 0.59, 1.08 | 0.12 | <.001 |
|  |  |  |  |  |  |  |  |  |  |  |
| σ^2^ | 0.95 |  |  |  |  | 0.98 |  |  |  |  |
| τ_00_ _Class_ | 0.02 |  |  |  |  | 0.00 |  |  |  |  |
| ICC | 0.02 |  |  |  |  | 0.00 |  |  |  |  |
| N_Class_ | 10 |  |  |  |  | 10 |  |  |  |  |
| Observations | 378 |  |  |  |  | 378 |  |  |  |  |
| Marginal R^2^ | .138 |  |  |  |  | .127 |  |  |  |  |
|  | *Positive Uniting* | | | | | | | | | |
|  | *β* | *b* | *95% CI* | *SE* | *p* | *β* | *b* | *95% CI* | *SE* | *p* |
| (Intercept) | 0.13 | 2.48 | 1.76, 3.21 | 0.37 | <.001 | 0.09 | 1.63 | 0.61, 2.65 | 0.52 | .002 |
| BSDS | 0.09 | 0.43 | -0.03, 0.89 | 0.23 | .068 | 0.10 | 0.47 | 0.00, 0.93 | 0.24 | .048 |
| GIHS | 0.31 | 0.56 | 0.39, 0.73 | 0.09 | <.001 |  |  |  |  |  |
| CIHS |  |  |  |  |  | 0.29 | 0.80 | 0.54, 1.06 | 0.13 | <.001 |
|  |  |  |  |  |  |  |  |  |  |  |
| σ^2^ | 1.08 |  |  |  |  | 1.10 |  |  |  |  |
| τ_00_ _Class_ | 0.09 |  |  |  |  | 0.06 |  |  |  |  |
| ICC | 0.08 |  |  |  |  | 0.05 |  |  |  |  |
| N_Class_ | 10 |  |  |  |  | 10 |  |  |  |  |
| Observations | 378 |  |  |  |  | 378 |  |  |  |  |
| Marginal R^2^ | .107 |  |  |  |  | .096 |  |  |  |  |
|  | *Positive Composite* | | | | | | | | | |
|  | *β* | *b* | *95% CI* | *SE* | *p* | *β* | *b* | *95% CI* | *SE* | *p* |
| (Intercept) | 0.09 | 2.60 | 2.04, 3.17 | 0.29 | <.001 | 0.04 | 2.03 | 1.21, 2.84 | 0.41 | <.001 |
| BSDS | 0.09 | 0.35 | -0.02, 0.71 | 0.19 | .065 | 0.10 | 0.39 | 0.01, 0.76 | 0.19 | .042 |
| GIHS | 0.36 | 0.51 | 0.37, 0.65 | 0.07 | <.001 |  |  |  |  |  |
| CIHS |  |  |  |  |  | 0.31 | 0.68 | 0.46, 0.89 | 0.11 | <.001 |
|  |  |  |  |  |  |  |  |  |  |  |
| σ^2^ | 0.69 |  |  |  |  | 0.73 |  |  |  |  |
| τ_00_ _Class_ | 0.03 |  |  |  |  | 0.01 |  |  |  |  |
| ICC | 0.04 |  |  |  |  | 0.01 |  |  |  |  |
| N_Class_ | 10 |  |  |  |  | 10 |  |  |  |  |
| Observations | 378 |  |  |  |  | 378 |  |  |  |  |
| Marginal R^2^ | .138 |  |  |  |  | .107 |  |  |  |  |
|  | *Negative Attacking* | | | | | | | | | |
|  | *β* | *b* | *95% CI* | *SE* | *p* | *β* | *b* | *95% CI* | *SE* | *p* |
| (Intercept) | 0.00 | 4.26 | 3.59, 4.93 | 0.34 | <.001 | 0.00 | 5.85 | 4.91, 6.79 | 0.48 | <.001 |
| BSDS | -0.11 | -0.50 | -0.96, -0.05 | 0.23 | .029 | -0.11 | -0.48 | -0.92, -0.04 | 0.22 | .034 |
| GIHS | -0.15 | -0.24 | -0.41, -0.08 | 0.08 | .004 |  |  |  |  |  |
| CIHS |  |  |  |  |  | -0.27 | -0.69 | -0.93, -0.44 | 0.13 | <.001 |
|  |  |  |  |  |  |  |  |  |  |  |
| Observations | 378 |  |  |  |  | 378 |  |  |  |  |
| R^2^ | .037 |  |  |  |  | .087 |  |  |  |  |
|  | *Negative Evading* | | | | | | | | | |
|  | *β* | *b* | *95% CI* | *SE* | *p* | *β* | *b* | *95% CI* | *SE* | *p* |
| (Intercept) | -0.05 | 3.75 | 2.94, 4.56 | 0.41 | <.001 | -0.05 | 4.67 | 3.51, 5.82 | 0.59 | <.001 |
| BSDS | -0.14 | -0.75 | -1.28, -0.22 | 0.27 | .006 | -0.13 | -0.71 | -1.24, -0.18 | 0.27 | .009 |
| GIHS | 0.05 | 0.09 | -0.11, 0.28 | 0.10 | .371 |  |  |  |  |  |
| CIHS |  |  |  |  |  | -0.05 | -0.16 | -0.46, 0.15 | 0.15 | .311 |
|  |  |  |  |  |  |  |  |  |  |  |
| σ^2^ | 1.46 |  |  |  |  | 1.46 |  |  |  |  |
| τ_00_ _Class_ | 0.03 |  |  |  |  | 0.03 |  |  |  |  |
| ICC | 0.02 |  |  |  |  | 0.02 |  |  |  |  |
| N_Class_ | 10 |  |  |  |  | 10 |  |  |  |  |
| Observations | 378 |  |  |  |  | 378 |  |  |  |  |
| Marginal R^2^ | .021 |  |  |  |  | .021 |  |  |  |  |
|  | *Negative Composite* | | | | | | | | | |
|  | *β* | *b* | *95% CI* | *SE* | *p* | *β* | *b* | *95% CI* | *SE* | *p* |
| (Intercept) | -0.00 | 4.09 | 3.54, 4.63 | 0.28 | <.001 | -0.00 | 5.45 | 4.69, 6.21 | 0.39 | <.001 |
| BSDS | -0.16 | -0.59 | -0.95, -0.22 | 0.19 | .002 | -0.15 | -0.55 | -0.91, -0.20 | 0.18 | .002 |
| GIHS | -0.09 | -0.13 | -0.26, 0.01 | 0.07 | .065 |  |  |  |  |  |
| CIHS |  |  |  |  |  | -0.24 | -0.50 | -0.70, -0.30 | 0.10 | <.001 |
|  |  |  |  |  |  |  |  |  |  |  |
| Observations | 378 |  |  |  |  | 378 |  |  |  |  |
| Marginal R^2^ | .037 |  |  |  |  | .087 |  |  |  |  |

*Note:* The impact of GIHS on the negative composite becomes significant (*p* = .022) after removing outliers on the negative composite measure.

**Analyses Controlling for Gender and Age**

We entered age and gender into each model as a robustness check in both studies. We asked participants, “How do you describe your gender?” with answer options of “female,” “male,” “non-binary,” “other (please specify),” and “prefer not to say.” We dummy coded gender such that “female” was the comparison group (0) and all other identities were separate dummy variables (1’s). Because of the small frequencies of participants identifying as nonbinary or who chose to self-report in this sample, we recommend caution when generalizing these coefficients.

**S6 Table. Regression models using intellectual humility as a predictor controlling for gender and age, Study 1**

|  | *Positive Informing* | | | | | | | | | |
| --- | --- | --- | --- | --- | --- | --- | --- | --- | --- | --- |
|  | *β* | *b* | *95% CI* | *SE* | *p* | *β* | *b* | *95% CI* | *SE* | *p* |
| (Intercept) | 0.00 | 3.30 | 2.38, 4.22 | 0.47 | <.001 | 0.00 | 3.67 | 2.50, 4.84 | 0.59 | <.001 |
| Age | 0.05 | 0.02 | -0.02, 0.05 | 0.02 | .319 | 0.06 | 0.02 | -0.01, 0.05 | 0.02 | .296 |
| Gender Male | -0.02 | -0.05 | -0.29, 0.19 | 0.12 | .673 | -0.03 | -0.07 | -0.32, 0.18 | 0.13 | .586 |
| Gender Nonbinary | 0.01 | 0.07 | -0.46, 0.60 | 0.27 | .793 | 0.03 | 0.15 | -0.39, 0.68 | 0.27 | .594 |
| Gender Self-report | -0.09 | -1.05 | -2.24, 0.14 | 0.60 | .083 | -0.07 | -0.82 | -2.03, 0.39 | 0.61 | .184 |
| GIHS | 0.21 | 0.35 | 0.18, 0.52 | 0.09 | <.001 |  |  |  |  |  |
| CIHS |  |  |  |  |  | 0.10 | 0.26 | -0.01. 0.53 | 0.14 | .057 |
|  |  |  |  |  |  |  |  |  |  |  |
| Observations | 364 |  |  |  |  | 364 |  |  |  |  |
| R^2^ | .054 |  |  |  |  | .020 |  |  |  |  |
|  | *Positive Evading* | | | | | | | | | |
|  | *β* | *b* | *95% CI* | *SE* | *p* | *β* | *b* | *95% CI* | *SE* | *p* |
| (Intercept) | -0.00 | 2.88 | 1.83, 3.93 | 0.54 | <.001 | -0.00 | 2.85 | 1.52, 4.19 | 0.68 | <.001 |
| Age | -0.07 | -0.03 | -0.06, 0.01 | 0.02 | .148 | -0.07 | -0.03 | -0.06, 0.01 | 0.02 | .156 |
| Gender Male | -0.07 | -0.18 | -0.46, 0.10 | 0.14 | .202 | -0.08 | -0.22 | -0.50, 0.07 | 0.14 | .134 |
| Gender Nonbinary | 0.07 | 0.44 | -0.16, 1.05 | 0.31 | .151 | 0.09 | 0.54 | -0.07, 1.15 | 0.31 | .082 |
| Gender Self-report | 0.00 | 0.00 | -1.36, 1.37 | 0.69 | .996 | 0.02 | 0.33 | -1.05, 1.71 | 0.70 | .638 |
| GIHS | 0.23 | 0.43 | 0.24, 0.63 | 0.10 | <.001 |  |  |  |  |  |
| CIHS |  |  |  |  |  | 0.16 | 0.46 | 0.16, 0.77 | 0.16 | .003 |
|  |  |  |  |  |  |  |  |  |  |  |
| Observations | 364 |  |  |  |  | 364 |  |  |  |  |
| R^2^ | .068 |  |  |  |  | .041 |  |  |  |  |
|  | *Positive Opening* | | | | | | | | | |
|  | *β* | *b* | *95% CI* | *SE* | *p* | *β* | *b* | *95% CI* | *SE* | *p* |
| (Intercept) | 0.07 | 2.39 | 1.50, 3.27 | 0.45 | <.001 | 0.01 | 1.67 | 0.56, 2.78 | 0.56 | .003 |
| Age | 0.02 | 0.01 | -0.02, 0.04 | 0.02 | .638 | 0.01 | 0.00 | -0.03, 0.03 | 0.02 | .840 |
| Gender Male | 0.08 | 0.19 | -0.04, 0.42 | 0.12 | .106 | 0.05 | 0.11 | -0.13, 0.34 | 0.12 | .376 |
| Gender Nonbinary | -0.02 | -0.09 | -0.59, 0.42 | 0.26 | .735 | 0.00 | 0.00 | -0.51, 0.51 | 0.26 | .995 |
| Gender Self-report | -0.05 | -0.62 | -1.74, 0.51 | 0.57 | .280 | -0.01 | -0.16 | -1.31, 0.98 | 0.58 | .780 |
| GIHS | 0.36 | 0.59 | 0.43, 0.75 | 0.08 | <.001 |  |  |  |  |  |
| CIHS |  |  |  |  |  | 0.32 | 0.82 | 0.57, 1.08 | 0.13 | <.001 |
|  |  |  |  |  |  |  |  |  |  |  |
| σ^2^ | 0.96 |  |  |  |  | 1.00 |  |  |  |  |
| τ_00_ _Class_ | 0.03 |  |  |  |  | 0.00 |  |  |  |  |
| ICC | 0.03 |  |  |  |  | 0.00 |  |  |  |  |
| N_Class_ | 10 |  |  |  |  | 10 |  |  |  |  |
| Observations | 365 |  |  |  |  | 365 |  |  |  |  |
| Marginal R^2^ | .132 |  |  |  |  | .108 |  |  |  |  |
|  | *Positive Uniting* | | | | | | | | | |
|  | *β* | *b* | *95% CI* | *SE* | *p* | *β* | *b* | *95% CI* | *SE* | *p* |
| (Intercept) | 0.15 | 2.25 | 1.29, 3.21 | 0.49 | <.001 | 0.12 | 1.61 | 0.42, 2.81 | 0.61 | .008 |
| Age | 0.06 | 0.02 | -0.01, 0.05 | 0.02 | .262 | 0.05 | 0.01 | -0.02, 0.05 | 0.02 | .369 |
| Gender Male | 0.07 | 0.17 | -0.08, 0.41 | 0.12 | .175 | 0.04 | 0.10 | -0.15, 0.34 | 0.13 | .453 |
| Gender Nonbinary | 0.00 | 0.01 | -0.52, 0.55 | 0.27 | .966 | 0.02 | 0.12 | -0.42, 0.66 | 0.28 | .665 |
| Gender Self-report | -0.03 | -0.38 | -1.57, 0.81 | 0.60 | .529 | 0.01 | 0.06 | -1.14, 1.27 | 0.61 | .918 |
| GIHS | 0.32 | 0.56 | 0.39, 0.73 | 0.09 | <.001 |  |  |  |  |  |
| CIHS |  |  |  |  |  | 0.28 | 0.77 | 0.50, 1.05 | 0.14 | <.001 |
|  |  |  |  |  |  |  |  |  |  |  |
| σ^2^ | 1.07 |  |  |  |  | 1.11 |  |  |  |  |
| τ_00_ _Class_ | 0.13 |  |  |  |  | 0.09 |  |  |  |  |
| ICC | 0.11 |  |  |  |  | 0.07 |  |  |  |  |
| N_Class_ | 10 |  |  |  |  | 10 |  |  |  |  |
| Observations | 365 |  |  |  |  | 365 |  |  |  |  |
| Marginal R^2^ | .104 |  |  |  |  | .085 |  |  |  |  |
|  | *Positive Composite* | | | | | | | | | |
|  | *β* | *b* | *95% CI* | *SE* | *p* | *β* | *b* | *95% CI* | *SE* | *p* |
| (Intercept) | 0.10 | 2.58 | 1.82, 3.33 | 0.39 | <.001 | 0.05 | 2.15 | 1.19, 3.11 | 0.49 | <.001 |
| Age | 0.02 | 0.01 | -0.02, 0.03 | 0.01 | .647 | 0.01 | 0.00 | -0.02, 0.03 | 0.01 | .805 |
| Gender Male | 0.04 | 0.08 | -0.11, 0.28 | 0.10 | .404 | 0.01 | 0.02 | -0.18, 0.22 | 0.10 | .854 |
| Gender Nonbinary | 0.01 | 0.06 | -0.37, 0.49 | 0.22 | .796 | 0.03 | 0.14 | -0.30, 0.59 | 0.22 | .520 |
| Gender Self-report | -0.05 | -0.50 | -1.46, 0.46 | 0.49 | .305 | -0.01 | -0.11 | -1.09, 0.88 | 0.50 | .831 |
| GIHS | 0.37 | 0.52 | 0.38, 0.66 | 0.07 | <.001 |  |  |  |  |  |
| CIHS |  |  |  |  |  | 0.30 | 0.67 | 0.45, 0.89 | 0.11 | <.001 |
|  |  |  |  |  |  |  |  |  |  |  |
| σ^2^ | 0.70 |  |  |  |  | 0.74 |  |  |  |  |
| τ_00_ _Class_ | 0.03 |  |  |  |  | 0.01 |  |  |  |  |
| ICC | 0.04 |  |  |  |  | 0.02 |  |  |  |  |
| N_Class_ | 10 |  |  |  |  | 10 |  |  |  |  |
| Observations | 365 |  |  |  |  | 365 |  |  |  |  |
| Marginal R^2^ | .133 |  |  |  |  | .092 |  |  |  |  |
|  | *Negative Attacking* | | | | | | | | | |
|  | *β* | *b* | *95% CI* | *SE* | *p* | *β* | *b* | *95% CI* | *SE* | *p* |
| (Intercept) | -0.00 | 4.57 | 3.68, 5.47 | 0.46 | <.001 | 0.00 | 6.04 | 4.95, 7.13 | 0.56 | <.001 |
| Age | -0.08 | -0.02 | -0.05, 0.01 | 0.02 | .124 | -0.07 | -0.02 | -0.05, 0.01 | 0.02 | .184 |
| Gender Male | -0.02 | -0.04 | -0.28, 0.20 | 0.12 | .746 | 0.01 | 0.03 | -0.21, 0.26 | 0.12 | .820 |
| Gender Nonbinary | 0.02 | 0.11 | -0.41, 0.62 | 0.26 | .679 | 0.01 | 0.03 | -0.47, 0.53 | 0.25 | .911 |
| Gender Self-report | -0.06 | -0.69 | -1.85, 0.47 | 0.59 | .242 | -0.09 | -0.99 | -2.12, 0.14 | 0.57 | .086 |
| GIHS | -0.15 | -0.25 | -0.41, -0.08 | 0.08 | .003 |  |  |  |  |  |
| CIHS |  |  |  |  |  | -0.27 | -0.68 | -0.93, -0.43 | 0.13 | <.001 |
|  |  |  |  |  |  |  |  |  |  |  |
| Observations | 365 |  |  |  |  | 365 |  |  |  |  |
| R^2^ | .037 |  |  |  |  | .085 |  |  |  |  |
|  | *Negative Evading* | | | | | | | | | |
|  | *β* | *b* | *95% CI* | *SE* | *p* | *β* | *b* | *95% CI* | *SE* | *p* |
| (Intercept) | -0.06 | 3.89 | 2.82, 4.95 | 0.54 | <.001 | -0.06 | 4.48 | 3.15, 5.81 | 0.68 | <.001 |
| Age | -0.04 | -0.01 | -0.05, 0.02 | 0.02 | .444 | -0.03 | -0.01 | -0.05, 0.02 | 0.02 | .511 |
| Gender Male | -0.19 | -0.52 | -0.80, -0.24 | 0.14 | <.001 | -0.19 | -0.51 | -0.79, -0.23 | 0.14 | <.001 |
| Gender Nonbinary | 0.06 | 0.36 | -0.25, 0.97 | 0.31 | .246 | 0.06 | 0.37 | -0.24, 0.98 | 0.31 | .238 |
| Gender Self-report | 0.08 | 1.04 | -0.32, 2.41 | 0.69 | .134 | 0.08 | 1.06 | -0.31, 2.42 | 0.69 | .128 |
| GIHS | 0.04 | 0.08 | -0.12, 0.27 | 0.10 | .424 |  |  |  |  |  |
| CIHS |  |  |  |  |  | -0.03 | -0.09 | -0.39, 0.22 | 0.16 | .579 |
|  |  |  |  |  |  |  |  |  |  |  |
| σ^2^ | 1.42 |  |  |  |  | 1.42 |  |  |  |  |
| τ_00_ _Class_ | 0.03 |  |  |  |  | 0.03 |  |  |  |  |
| ICC | 0.02 |  |  |  |  | 0.02 |  |  |  |  |
| N_Class_ | 10 |  |  |  |  | 10 |  |  |  |  |
| Observations | 365 |  |  |  |  | 365 |  |  |  |  |
| Marginal R^2^ | .054 |  |  |  |  | .053 |  |  |  |  |
|  | *Negative Composite* | | | | | | | | | |
|  | *β* | *b* | *95% CI* | *SE* | *p* | *β* | *b* | *95% CI* | *SE* | *p* |
| (Intercept) | -0.00 | 4.32 | 3.58, 5.06 | 0.38 | <.001 | 0.00 | 5.50 | 4.60, 6.41 | 0.46 | <.001 |
| Age | -0.08 | -0.02 | -0.04, 0.01 | 0.01 | .123 | -0.07 | -0.02 | -0.04, 0.01 | 0.01 | .182 |
| Gender Male | -0.10 | -0.19 | -0.39, 0.00 | 0.10 | .051 | -0.08 | -0.15 | -0.34, 0.05 | 0.10 | .134 |
| Gender Nonbinary | 0.05 | 0.22 | -0.21, 0.64 | 0.22 | .319 | 0.04 | 0.17 | -0.25, 0.58 | 0.21 | .430 |
| Gender Self-report | -0.01 | -0.10 | -1.06, 0.86 | 0.49 | .837 | -0.03 | -0.29 | -1.22, 0.65 | 0.48 | .543 |
| GIHS | -0.10 | -0.13 | -0.27, 0.00 | 0.07 | .057 |  |  |  |  |  |
| CIHS |  |  |  |  |  | -0.23 | -0.48 | -0.68, -0.27 | 0.11 | <.001 |
|  |  |  |  |  |  |  |  |  |  |  |
| Observations | 365 |  |  |  |  | 365 |  |  |  |  |
| Marginal R^2^ | .033 |  |  |  |  | .075 |  |  |  |  |

*Note:* The impact of GIHS on the negative composite becomes significant (*p* = .018) after removing outliers on the negative composite measure.

**Additional Analyses: Study 2**

**S7 Table. Regression models using intellectual humility as a predictor on all conflict subscales, Study 2**

|  | *Positive Informing (Family)* | | | | | | | | | |
| --- | --- | --- | --- | --- | --- | --- | --- | --- | --- | --- |
|  | *β* | *b* | *95% CI* | *SE* | *p* | *β* | *b* | *95% CI* | *SE* | *p* |
| (Intercept) | 0.00 | 3.15 | 2.30, 3.99 | .43 | <.001 | 0.00 | 2.08 | 0.29, 3.87 | .91 | .023 |
| GIHS | 0.31 | 0.51 | 0.28, 0.75 | .12 | <.001 |  |  |  |  |  |
| CIHS |  |  |  |  |  | 0.24 | 0.76 | 0.29, 1.22 | .24 | .002 |
|  |  |  |  |  |  |  |  |  |  |  |
| Observations | 173 |  |  |  |  | 173 |  |  |  |  |
| R^2^ | .099 |  |  |  |  | .057 |  |  |  |  |
|  | *Positive Evading (Family)* | | | | | | | | | |
|  | *β* | *b* | *95% CI* | *SE* | *p* | *β* | *b* | *95% CI* | *SE* | *p* |
| (Intercept) | 0.00 | 3.34 | 2.51, 4.17 | .42 | <.001 | 0.00 | 3.89 | 2.15, 5.63 | .88 | <.001 |
| GIHS | 0.18 | 0.28 | 0.05, 0.50 | .12 | .019 |  |  |  |  |  |
| CIHS |  |  |  |  |  | 0.04 | 0.11 | -0.34, 0.56 | .23 | .624 |
|  |  |  |  |  |  |  |  |  |  |  |
| Observations | 173 |  |  |  |  | 173 |  |  |  |  |
| R^2^ | .032 |  |  |  |  | .001 |  |  |  |  |
|  | *Positive Opening (Family)* | | | | | | | | | |
|  | *β* | *b* | *95% CI* | *SE* | *p* | *β* | *b* | *95% CI* | *SE* | *p* |
| (Intercept) | -0.00 | 2.68 | 1.88, 3.48 | .41 | <.001 | -0.00 | 0.96 | -0.71, 2.64 | .85 | .258 |
| GIHS | 0.34 | 0.54 | 0.31, 0.76 | .11 | <.001 |  |  |  |  |  |
| CIHS |  |  |  |  |  | 0.31 | 0.95 | 0.51, 1.38 | .22 | <.001 |
|  |  |  |  |  |  |  |  |  |  |  |
| Observations | 173 |  |  |  |  | 173 |  |  |  |  |
| R^2^ | .117 |  |  |  |  | .097 |  |  |  |  |
|  | *Positive Uniting (Family)* | | | | | | | | | |
|  | *β* | *b* | *95% CI* | *SE* | *p* | *β* | *b* | *95% CI* | *SE* | *p* |
| (Intercept) | -0.00 | 3.22 | 2.48, 3.95 | .37 | <.001 | -0.00 | 1.94 | 0.39, 3.49 | .79 | .014 |
| GIHS | 0.34 | 0.49 | 0.29, 0.69 | .10 | <.001 |  |  |  |  |  |
| CIHS |  |  |  |  |  | 0.28 | .79 | 0.38, 1.19 | .20 | <.001 |
|  |  |  |  |  |  |  |  |  |  |  |
| Observations | 173 |  |  |  |  | 173 |  |  |  |  |
| R^2^ | .116 |  |  |  |  | .080 |  |  |  |  |
|  | *Negative Attacking (Family)* | | | | | | | | | |
|  | *β* | *b* | *95% CI* | *SE* | *p* | *β* | *b* | *95% CI* | *SE* | *p* |
| (Intercept) | -0.00 | 4.24 | 3.30, 5.18 | .48 | <.001 | -0.00 | 6.42 | 4.51, 8.34 | .97 | <.001 |
| GIHS | -0.17 | -0.30 | -0.56, -0.04 | .13 | .025 |  |  |  |  |  |
| CIHS |  |  |  |  |  | -0.25 | -0.85 | -1.35, -0.35 | .25 | <.001 |
|  |  |  |  |  |  |  |  |  |  |  |
| Observations | 173 |  |  |  |  | 173 |  |  |  |  |
| R^2^ | .029 |  |  |  |  | .062 |  |  |  |  |
|  | *Negative Evading (Family)* | | | | | | | | | |
|  | *β* | *b* | *95% CI* | *SE* | *p* | *β* | *b* | *95% CI* | *SE* | *p* |
| (Intercept) | -0.00 | 5.19 | 4.13, 6.25 | .54 | <.001 | -0.00 | 7.51 | 5.35, 9.66 | 1.09 | <.001 |
| GIHS | -0.16 | -0.31 | -0.60, -0.02 | .15 | .039 |  |  |  |  |  |
| CIHS |  |  |  |  |  | -0.23 | -0.89 | -1.45, -0.33 | .28 | .002 |
|  |  |  |  |  |  |  |  |  |  |  |
| Observations | 173 |  |  |  |  | 173 |  |  |  |  |
| R^2^ | .025 |  |  |  |  | .055 |  |  |  |  |
|  | *Positive Informing (Work)* | | | | | | | | | |
|  | *β* | *b* | *95% CI* | *SE* | *p* | *β* | *b* | *95% CI* | *SE* | *p* |
| (Intercept) | -0.09 | 3.76 | 3.00, 4.52 | .38 | <.001 | -0.08 | 2.26 | 0.82, 3.70 | .73 | .002 |
| GIHS | 0.27 | 0.37 | 0.17, 0.58 | .10 | <.001 |  |  |  |  |  |
| CIHS |  |  |  |  |  | 0.29 | 0.74 | 0.37, 1.11 | .19 | <.001 |
|  |  |  |  |  |  |  |  |  |  |  |
| σ^2^ | 0.59 |  |  |  |  | 0.58 |  |  |  |  |
| τ_00_ _AreaDept_ | 0.03 |  |  |  |  | 0.03 |  |  |  |  |
| ICC | 0.05 |  |  |  |  | 0.04 |  |  |  |  |
| N _AreaDept_ | 5 |  |  |  |  | 5 |  |  |  |  |
| Observations | 160 |  |  |  |  | 160 |  |  |  |  |
| Marginal R^2^ | .073 |  |  |  |  | .085 |  |  |  |  |
|  | *Positive Evading (Work)* | | | | | | | | | |
|  | *β* | *b* | *95% CI* | *SE* | *p* | *β* | *b* | *95% CI* | *SE* | *p* |
| (Intercept) | -0.08 | 4.02 | 3.16, 4.87 | .43 | <.001 | -0.08 | 3.96 | 2.32, 5.59 | .83 | <.001 |
| GIHS | 0.08 | 0.11 | -0.12, 0.35 | .12 | .329 |  |  |  |  |  |
| CIHS |  |  |  |  |  | 0.04 | 0.12 | -0.30, 0.54 | .21 | .570 |
|  |  |  |  |  |  |  |  |  |  |  |
| σ^2^ | 0.75 |  |  |  |  | 0.75 |  |  |  |  |
| τ_00_ _AreaDept_ | 0.03 |  |  |  |  | 0.03 |  |  |  |  |
| ICC | 0.04 |  |  |  |  | 0.04 |  |  |  |  |
| N _AreaDept_ | 5 |  |  |  |  | 5 |  |  |  |  |
| Observations | 160 |  |  |  |  | 160 |  |  |  |  |
| Marginal R^2^ | .006 |  |  |  |  | .002 |  |  |  |  |
|  | *Positive Opening (Work)* | | | | | | | | | |
|  | *β* | *b* | *95% CI* | *SE* | *p* | *β* | *b* | *95% CI* | *SE* | *p* |
| (Intercept) | -0.06 | 3.87 | 3.11, 4.64 | .39 | <.001 | -0.05 | 2.88 | 1.40, 4.36 | .75 | <.001 |
| GIHS | 0.28 | 0.38 | 0.17, 0.59 | .11 | <.001 |  |  |  |  |  |
| CIHS |  |  |  |  |  | 0.24 | 0.61 | 0.23, 0.99 | .19 | .002 |
|  |  |  |  |  |  |  |  |  |  |  |
| σ^2^ | 0.60 |  |  |  |  | 0.62 |  |  |  |  |
| τ_00_ _AreaDept_ | 0.03 |  |  |  |  | 0.03 |  |  |  |  |
| ICC | 0.04 |  |  |  |  | 0.04 |  |  |  |  |
| N _AreaDept_ | 5 |  |  |  |  | 5 |  |  |  |  |
| Observations | 160 |  |  |  |  | 160 |  |  |  |  |
| Marginal R^2^ | .076 |  |  |  |  | .057 |  |  |  |  |
|  | *Positive Uniting (Work)* | | | | | | | | | |
|  | *β* | *b* | *95% CI* | *SE* | *p* | *β* | *b* | *95% CI* | *SE* | *p* |
| (Intercept) | -0.12 | 3.94 | 3.28, 4.59 | .33 | <.001 | -0.09 | 3.20 | 1.93, 4.47 | .64 | <.001 |
| GIHS | 0.36 | 0.43 | 0.25, 0.60 | .09 | <.001 |  |  |  |  |  |
| CIHS |  |  |  |  |  | 0.27 | 0.59 | 0.26, 0.92 | .16 | <.001 |
|  |  |  |  |  |  |  |  |  |  |  |
| σ^2^ | 0.42 |  |  |  |  | 0.45 |  |  |  |  |
| τ_00_ _AreaDept_ | 0.04 |  |  |  |  | 0.04 |  |  |  |  |
| ICC | 0.09 |  |  |  |  | 0.07 |  |  |  |  |
| N _AreaDept_ | 5 |  |  |  |  | 5 |  |  |  |  |
| Observations | 160 |  |  |  |  | 160 |  |  |  |  |
| Marginal R^2^ | .122 |  |  |  |  | .070 |  |  |  |  |
|  | *Negative Attacking (Work)* | | | | | | | | | |
|  | *β* | *b* | *95% CI* | *SE* | *p* | *β* | *b* | *95% CI* | *SE* | *p* |
| (Intercept) | 0.00 | 2.63 | 1.91, 3.35 | .37 | <.001 | -0.00 | 5.32 | 3.98, 6.66 | .68 | <.001 |
| GIHS | -0.13 | -0.17 | -0.37, 0.03 | .10 | .096 |  |  |  |  |  |
| CIHS |  |  |  |  |  | -0.36 | -0.86 | -1.20, -0.51 | .18 | <.001 |
|  |  |  |  |  |  |  |  |  |  |  |
| Observations | 161 |  |  |  |  | 161 |  |  |  |  |
| R^2^ | .017 |  |  |  |  | .130 |  |  |  |  |
|  | *Negative Evading (Work)* | | | | | | | | | |
|  | *β* | *b* | *95% CI* | *SE* | *p* | *β* | *b* | *95% CI* | *SE* | *p* |
| (Intercept) | 0.03 | 4.06 | 3.01, 5.11 | .53 | <.001 | 0.02 | 6.69 | 4.71, 8.66 | 1.00 | <.001 |
| GIHS | -0.09 | -0.17 | -0.45, 0.12 | .15 | .253 |  |  |  |  |  |
| CIHS |  |  |  |  |  | -0.25 | -0.84 | -1.35, -0.33 | .26 | .001 |
|  |  |  |  |  |  |  |  |  |  |  |
| σ^2^ | 1.16 |  |  |  |  | 1.11 |  |  |  |  |
| τ_00_ _AreaDept_ | 0.03 |  |  |  |  | 0.01 |  |  |  |  |
| ICC | 0.02 |  |  |  |  | 0.01 |  |  |  |  |
| N _AreaDept_ | 5 |  |  |  |  | 5 |  |  |  |  |
| Observations | 160 |  |  |  |  | 160 |  |  |  |  |
| Marginal R^2^ | .008 |  |  |  |  | .062 |  |  |  |  |

**S8 Table. ICCs for empty models, Study 2**

| *Outcome* | *Department ICC* |
| --- | --- |
| Positive Inform (Family) | .000 |
| Positive Evade (Family) | .000 |
| Positive Open (Family) | .000 |
| Positive Unite (Family) | .000 |
| Positive Composite (Family) | .000 |
| Negative Attack (Family) | .000 |
| Negative Evading (Family) | .000 |
| Negative Composite (Family) | .000 |
| Positive Inform (Work) | .049 |
| Positive Evade (Work) | .041 |
| Positive Open (Work) | .045 |
| Positive Unite (Work) | .079 |
| Positive Composite (Work) | .088 |
| Negative Attack (Work) | .006 |
| Negative Evading (Work) | .029 |
| Negative Composite (Work) | .023 |

**S9 Table. Model Fit Statistics, Study 2**

| Outcome | Model Type | AIC | BIC | Log Likelihood | χ^2^ test against empty model (df in parentheses) |
| --- | --- | --- | --- | --- | --- |
| Positive Inform (Work) | Empty Model | 390.02 | 399.24 | -192.01 |  |
|  | Including GIHS | 379.43 | 391.74 | -185.72 | 12.58 (1) ^***^ |
| Positive Inform (Work) | Empty Model | 390.02 | 399.24 | -192.01 |  |
|  | Including CIHS | 377.13 | 289.43 | -184.56 | 14.89 (1) ^***^ |
| Positive Evade (Work) | Empty Model | 415.67 | 424.90 | -204.84 |  |
|  | Including GIHS | 416.69 | 428.99 | -204.34 | .98 (1) |
| Positive Evade (Work) | Empty Model | 415.67 | 424.90 | -204.84 |  |
|  | Including CIHS | 417.32 | 429.62 | -204.66 | .35 (1) |
| Positive Open (Work) | Empty Model | 394.21 | 403.43 | -194.10 |  |
|  | Including GIHS | 383.26 | 395.56 | -187.63 | 12.95 (1) ^***^ |
| Positive Open (Work) | Empty Model | 394.21 | 403.43 | -194.10 |  |
|  | Including CIHS | 386.38 | 398.68 | -189.19 | 9.83 (1) ^**^ |
| Positive Unite (Work) | Empty Model | 346.55 | 355.77 | -170.27 |  |
|  | Including GIHS | 326.35 | 338.66 | -159.18 | 22.19 (1) ^***^ |
| Positive Unite (Work) | Empty Model | 346.55 | 355.77 | -170.27 |  |
|  | Including CIHS | 336.03 | 348.33 | -164.01 | 12.52 (1) ^***^ |
| Positive Composite (Work) | Empty Model | 346.55 | 355.77 | -170.27 |  |
|  | Including GIHS | 298.52 | 310.82 | -145.26 | 50.03 (1) ^***^ |
| Positive Composite (Work) | Empty Model | 346.55 | 355.77 | -170.27 |  |
|  | Including CIHS | 304.07 | 316.37 | -148.04 | 44.48 (1) ^***^ |
| Negative Evading (Work) | Empty Model | 485.99 | 495.22 | -240.00 |  |
|  | Including GIHS | 486.51 | 498.81 | -239.25 | 1.48 (1) |
| Negative Evading (Work) | Empty Model | 485.99 | 495.22 | -240.00 |  |
|  | Including CIHS | 477.46 | 489.76 | -234.73 | 10.54 (1) ^**^ |
| Negative Composite (Work) | Empty Model | 353.65 | 362.88 | -173.83 |  |
|  | Including GIHS | 352.20 | 364.50 | -172.10 | 3.45 (1) |
| Negative Composite (Work) | Empty Model | 353.65 | 362.88 | -173.83 |  |
|  | Including CIHS | 329.97 | 342.27 | -160.99 | 25.68 (1) ^***^ |

**Conflict Emotions**

Participants completed the same emotion measures as in Study 1 while thinking about conflicts with a friend or family member (*α* _positive emotions_ = .78, *α* _negative emotions_ = .88) and while thinking about conflicts with a work supervisor and supervisee (*α* _positive emotions_ = .87, *α* _negative emotions_ = .93).

GIHS and CIHS predicted more positive conflict emotions with friends and family. Only CIHS predicted less negative conflict emotions with friends and family. GIHS but not CIHS predicted more positive conflict emotions. CIHS but not GIHS predicted less negative conflict emotions.

**S10 Table. Regression models using intellectual humility as a predictor of conflict emotions, Study 2**

|  | *Positive Emotions (Family)* | | | | | | | | | |
| --- | --- | --- | --- | --- | --- | --- | --- | --- | --- | --- |
|  | *β* | *b* | *CI* | *SE* | *p* | *β* | *b* | *CI* | *SE* | *p* |
| (Intercept) | -0.00 | 1.30 | 0.31, 2.28 | .50 | .010 | -0.00 | 0.45 | -1.68, 2.58 | 1.08 | .677 |
| GIHS | 0.36 | 0.70 | 0.42, 0.97 | .14 | <.001 |  |  |  |  |  |
| CIHS |  |  |  |  |  | 0.23 | 0.87 | 0.31, 1.42 | .28 | .002 |
|  |  |  |  |  |  |  |  |  |  |  |
| Observations | 172 |  |  |  |  | 172 |  |  |  |  |
| R^2^ | .130 |  |  |  |  | .053 |  |  |  |  |
|  | *Positive Emotions (Work)* | | | | | | | | | |
|  | *β* | *b* | *CI* | *SE* | *p* | *β* | *b* | *CI* | *SE* | *p* |
| (Intercept) | -0.03 | 2.91 | 1.81, 4.01 | .56 | <.001 | -0.03 | 3.02 | 0.89, 5.12 | 1.08 | .006 |
| GIHS | 0.19 | 0.37 | 0.07, 0.66 | .15 | .016 |  |  |  |  |  |
| CIHS |  |  |  |  |  | 0.09 | 0.31 | -0.24, 0.86 | .28 | .266 |
|  |  |  |  |  |  |  |  |  |  |  |
| σ^2^ | 1.23 |  |  |  |  | 1.26 |  |  |  |  |
| τ_00_ _AreaDept_ | 0.06 |  |  |  |  | 0.08 |  |  |  |  |
| ICC | 0.05 |  |  |  |  | 0.06 |  |  |  |  |
| N_AreaDept_ | 5 |  |  |  |  | 5 |  |  |  |  |
| Observations | 160 |  |  |  |  | 160 |  |  |  |  |
| Marginal R^2^ | .035 |  |  |  |  | .007 |  |  |  |  |
|  | *Negative Emotions (Family)* | | | | | | | | | |
|  | *β* | *b* | *CI* | *SE* | *p* | *β* | *b* | *CI* | *SE* | *p* |
| (Intercept) | -0.00 | 4.28 | 3.26, 5.31 | .52 | <.001 | -0.00 | 6.16 | 4.05, 8.26 | 1.07 | <.001 |
| GIHS | -0.11 | -0.21 | -0.49, 0.08 | .14 | .150 |  |  |  |  |  |
| CIHS |  |  |  |  |  | -0.19 | -0.68 | -1.23, -0.13 | .28 | .015 |
|  |  |  |  |  |  |  |  |  |  |  |
| Observations | 172 |  |  |  |  | 172 |  |  |  |  |
| R^2^ | .012 |  |  |  |  | .034 |  |  |  |  |
|  | *Negative Emotions (Work)* | | | | | | | | | |
|  | *β* | *b* | *CI* | *SE* | *p* | *β* | *b* | *CI* | *SE* | *p* |
| (Intercept) | -0.01 | 2.15 | 1.16, 3.14 | .50 | <.001 | 0.00 | 4.93 | 3.06, 6.79 | .95 | <.001 |
| GIHS | 0.05 | 0.08 | -0.19, 0.35 | .14 | .537 |  |  |  |  |  |
| CIHS |  |  |  |  |  | -0.20 | -0.64 | -1.12, -0.16 | .24 | .010 |
|  |  |  |  |  |  |  |  |  |  |  |
| σ^2^ | 1.02 |  |  |  |  | — |  |  |  |  |
| τ_00_ _AreaDept_ | 0.02 |  |  |  |  | — |  |  |  |  |
| ICC | 0.02 |  |  |  |  | — |  |  |  |  |
| N_AreaDept_ | 5 |  |  |  |  | — |  |  |  |  |
| Observations | 160 |  |  |  |  | 160 |  |  |  |  |
| Marginal R^2^ | .002 |  |  |  |  | .041 |  |  |  |  |

**Analyses Controlling for Gender and Age**

**S11 Table. Regression models using intellectual humility as a predictor controlling for age and gender, Study 2**

|  | *Positive Informing (Family)* | | | | | | | | | |
| --- | --- | --- | --- | --- | --- | --- | --- | --- | --- | --- |
|  | *β* | *b* | *95% CI* | *SE* | *p* | *β* | *b* | *95% CI* | *SE* | *p* |
| (Intercept) | 0.00 | 3.69 | 2.40, 4.98 | 0.65 | <.001 | 0.00 | 2.91 | 0.93, 4.88 | 1.00 | .004 |
| Age | -0.05 | -0.01 | -0.02, 0.01 | 0.01 | .496 | -0.10 | -0.01 | -0.03, 0.01 | 0.01 | .182 |
| Gender Male | -0.04 | -0.10 | -0.45, 0.26 | 0.18 | .592 | -0.01 | -0.02 | -0.37, 0.34 | 0.18 | .920 |
| Gender Prefer Not to Say | 0.05 | 0.70 | -1.20, 2.60 | 0.96 | .469 | 0.08 | 1.06 | -0.88, 3.00 | 0.98 | .283 |
| GIHS | 0.28 | 0.45 | 0.20, 0.70 | 0.12 | <.001 |  |  |  |  |  |
| CIHS |  |  |  |  |  | 0.22 | 0.69 | 0.22, 1.16 | 0.24 | .005 |
|  |  |  |  |  |  |  |  |  |  |  |
| Observations | 168 |  |  |  |  | 168 |  |  |  |  |
| R^2^ | .086 |  |  |  |  | .060 |  |  |  |  |
|  | *Positive Evading (Family)* | | | | | | | | | |
|  | *β* | *b* | *95% CI* | *SE* | *p* | *β* | *b* | *95% CI* | *SE* | *p* |
| (Intercept) | -0.00 | 3.07 | 1.80, 4.33 | 0.64 | <.001 | -0.00 | 3.76 | 1.83, 5.70 | 0.98 | <.001 |
| Age | 0.05 | 0.01 | -0.01, 0.02 | 0.01 | .539 | 0.02 | 0.00 | -0.01, 0.02 | 0.01 | .833 |
| Gender Male | -0.04 | -0.08 | -0.43, 0.27 | 0.18 | .646 | -0.01 | -0.02 | -0.37, 0.32 | 0.18 | .892 |
| Gender Prefer Not to Say | 0.14 | 1.67 | -0.19, 3.53 | 0.94 | .078 | 0.14 | 1.74 | -0.17, 3.65 | 0.97 | .073 |
| GIHS | 0.18 | 0.29 | 0.05, 0.53 | 0.12 | .020 |  |  |  |  |  |
| CIHS |  |  |  |  |  | 0.04 | 0.12 | -0.34, 0.58 | 0.23 | .602 |
|  |  |  |  |  |  |  |  |  |  |  |
| Observations | 168 |  |  |  |  | 168 |  |  |  |  |
| R^2^ | .051 |  |  |  |  | .021 |  |  |  |  |
|  | *Positive Opening (Family)* | | | | | | | | | |
|  | *β* | *b* | *95% CI* | *SE* | *p* | *β* | *b* | *95% CI* | *SE* | *p* |
| (Intercept) | -0.00 | 2.68 | 1.45, 3.92 | 0.63 | <.001 | 0.00 | 1.34 | -0.54, 3.21 | 0.95 | .162 |
| Age | 0.01 | 0.00 | -0.01, 0.02 | 0.01 | .875 | -0.05 | -0.01 | -0.02, 0.01 | 0.01 | .524 |
| Gender Male | 0.03 | 0.07 | -0.27, 0.41 | 0.17 | .669 | 0.07 | 0.16 | -0.18, 0.50 | 0.17 | .353 |
| Gender Prefer Not to Say | -0.00 | -0.05 | -1.87, 1.78 | 0.92 | .960 | 0.03 | 0.43 | -1.42, 2.28 | 0.94 | .649 |
| GIHS | 0.33 | 0.51 | 0.28, 0.75 | 0.12 | <.001 |  |  |  |  |  |
| CIHS |  |  |  |  |  | 0.30 | 0.90 | 0.46, 1.35 | 0.23 | <.001 |
|  |  |  |  |  |  |  |  |  |  |  |
| Observations | 168 |  |  |  |  | 168 |  |  |  |  |
| R^2^ | .110 |  |  |  |  | .096 |  |  |  |  |
|  | *Positive Uniting (Family)* | | | | | | | | | |
|  | *β* | *b* | *95% CI* | *SE* | *p* | *β* | *b* | *95% CI* | *SE* | *p* |
| (Intercept) | -0.00 | 3.27 | 2.14, 4.41 | 0.58 | <.001 | 0.00 | 2.24 | 0.50, 3.98 | 0.88 | .012 |
| Age | 0.01 | 0.00 | -0.01, 0.02 | 0.01 | .943 | -0.05 | -0.01 | -0.02, 0.01 | 0.01 | .487 |
| Gender Male | 0.03 | 0.06 | -0.25, 0.37 | 0.16 | .697 | 0.07 | 0.14 | -0.17, 0.45 | 0.16 | .378 |
| Gender Prefer Not to Say | 0.07 | 0.80 | -0.88, 2.48 | 0.85 | .346 | 0.10 | 1.20 | -0.51, 2.92 | 0.87 | .167 |
| GIHS | 0.32 | 0.46 | 0.24, 0.68 | 0.11 | <.001 |  |  |  |  |  |
| CIHS |  |  |  |  |  | 0.27 | 0.76 | 0.35, 1.18 | 0.21 | <.001 |
|  |  |  |  |  |  |  |  |  |  |  |
| Observations | 168 |  |  |  |  | 168 |  |  |  |  |
| R^2^ | .109 |  |  |  |  | .086 |  |  |  |  |
|  | *Positive Composite (Family)* | | | | | | | | | |
|  | *β* | *b* | *95% CI* | *SE* | *p* | *β* | *b* | *95% CI* | *SE* | *p* |
| (Intercept) | -0.00 | 3.12 | 2.13, 4.10 | 0.50 | <.001 | 0.00 | 2.30 | 0.78, 3.82 | 0.77 | .003 |
| Age | 0.01 | 0.00 | -0.01, 0.01 | 0.01 | .939 | -0.06 | -0.00 | -0.02, 0.01 | 0.01 | .439 |
| Gender Male | 0.01 | 0.02 | -0.25, 0.29 | 0.14 | .895 | 0.05 | 0.10 | -0.18, 0.37 | 0.14 | .492 |
| Gender Prefer Not to Say | 0.07 | 0.66 | -0.80, 2.11 | 0.74 | .374 | 0.10 | 1.02 | -0.48, 2.52 | 0.76 | .180 |
| GIHS | 0.35 | 0.45 | 0.26, 0.64 | 0.09 | <.001 |  |  |  |  |  |
| CIHS |  |  |  |  |  | 0.29 | 0.69 | 0.33, 1.06 | 0.18 | <.001 |
|  |  |  |  |  |  |  |  |  |  |  |
| Observations | 168 |  |  |  |  | 168 |  |  |  |  |
| R^2^ | .129 |  |  |  |  | .090 |  |  |  |  |
|  | *Negative Attacking (Family)* | | | | | | | | | |
|  | *β* | *b* | *95% CI* | *SE* | *p* | *β* | *b* | *95% CI* | *SE* | *p* |
| (Intercept) | 0.00 | 4.31 | 2.86, 5.75 | 0.73 | <.001 | 0.00 | 6.32 | 4.18, 8.46 | 1.08 | <.001 |
| Age | -0.02 | -0.00 | -0.02, 0.02 | 0.01 | .846 | 0.01 | 0.00 | -0.02, 0.02 | 0.01 | .848 |
| Gender Male | -0.11 | -0.29 | -0.68, 0.11 | 0.20 | .156 | -0.13 | -0.32 | -0.71, 0.06 | 0.20 | .099 |
| Gender Prefer Not to Say | 0.04 | 0.62 | -1.51, 2.75 | 1.08 | .568 | 0.01 | 0.19 | -1.92, 2.30 | 1.07 | .857 |
| GIHS | -0.15 | -0.27 | -0.55, 0.00 | 0.14 | .052 |  |  |  |  |  |
| CIHS |  |  |  |  |  | -0.24 | -0.82 | -1.33, -0.31 | 0.26 | .002 |
|  |  |  |  |  |  |  |  |  |  |  |
| Observations | 168 |  |  |  |  | 168 |  |  |  |  |
| R^2^ | .043 |  |  |  |  | .077 |  |  |  |  |
|  | *Negative Evading (Family)* | | | | | | | | | |
|  | *β* | *b* | *95% CI* | *SE* | *p* | *β* | *b* | *95% CI* | *SE* | *p* |
| (Intercept) | 0.00 | 4.48 | 2.85, 6.10 | 0.82 | <.001 | 0.00 | 6.69 | 4.28, 9.10 | 1.22 | <.001 |
| Age | 0.10 | 0.01 | -0.01, 0.03 | 0.01 | .191 | 0.13 | 0.02 | -0.00, 0.04 | 0.01 | .084 |
| Gender Male | -0.02 | -0.05 | -0.50, 0.40 | 0.23 | .827 | -0.03 | -0.09 | -0.52, 0.34 | 0.22 | .680 |
| Gender Prefer Not to Say | 0.09 | 1.36 | -1.04, 3.76 | 1.22 | .266 | 0.06 | 0.89 | -1.49, 3.27 | 1.20 | .461 |
| GIHS | -0.15 | -0.30 | -0.61, 0.01 | 0.16 | .057 |  |  |  |  |  |
| CIHS |  |  |  |  |  | -0.24 | -0.90 | -1.48, -0.33 | 0.29 | .002 |
|  |  |  |  |  |  |  |  |  |  |  |
| Observations | 168 |  |  |  |  | 168 |  |  |  |  |
| R^2^ | .047 |  |  |  |  | .079 |  |  |  |  |
|  | *Negative Composite (Family)* | | | | | | | | | |
|  | *β* | *b* | *95% CI* | *SE* | *p* | *β* | *b* | *95% CI* | *SE* | *p* |
| (Intercept) | -0.00 | 4.36 | 3.18, 5.55 | 0.60 | <.001 | -0.00 | 6.44 | 4.70, 8.18 | 0.88 | <.001 |
| Age | 0.03 | 0.00 | -0.01, 0.02 | 0.01 | .659 | 0.07 | 0.01 | -0.01, 0.02 | 0.01 | .337 |
| Gender Male | -0.10 | -0.21 | -0.53, 0.12 | 0.16 | .210 | -0.11 | -0.25 | -0.56, 0.07 | 0.16 | .122 |
| Gender Prefer Not to Say | 0.07 | 0.86 | -0.88, 2.61 | 0.89 | .330 | 0.04 | 0.42 | -1.29, 2.14 | 0.87 | .625 |
| GIHS | -0.19 | -0.28 | -0.51, -0.06 | 0.11 | .015 |  |  |  |  |  |
| CIHS |  |  |  |  |  | -0.30 | -0.85 | -1.26, -0.43 | 0.21 | <.001 |
|  |  |  |  |  |  |  |  |  |  |  |
| Observations | 168 |  |  |  |  | 168 |  |  |  |  |
| R^2^ | .061 |  |  |  |  | .114 |  |  |  |  |
|  | *Positive Informing (Work)* | | | | | | | | | |
|  | *β* | *b* | *95% CI* | *SE* | *p* | *β* | *b* | *95% CI* | *SE* | *p* |
| (Intercept) | -0.08 | 2.96 | 1.83, 4.06 | 0.57 | <.001 | -0.08 | 1.84 | 0.26, 3.43 | 0.80 | .023 |
| Age | 0.16 | 0.01 | 0.00, 0.03 | 0.01 | .039 | 0.10 | 0.01 | -0.00, 0.02 | 0.01 | .193 |
| Gender Male | -0.04 | -0.08 | -0.36, 0.21 | 0.14 | .588 | -0.00 | -0.01 | -0.29, 0.27 | 0.14 | .964 |
| Gender Prefer Not to Say | 0.08 | 0.85 | -0.64, 2.34 | 0.75 | .264 | 0.13 | 1.26 | -0.23, 2.76 | 0.76 | .098 |
| GIHS | 0.30 | 0.40 | 0.19, 0.61 | 0.11 | <.001 |  |  |  |  |  |
| CIHS |  |  |  |  |  | 0.30 | 0.73 | 0.36, 1.10 | 0.19 | <.001 |
|  |  |  |  |  |  |  |  |  |  |  |
| σ^2^ | 0.56 |  |  |  |  | 0.55 |  |  |  |  |
| τ_00_ _AreaDept_ | 0.03 |  |  |  |  | 0.03 |  |  |  |  |
| ICC | 0.05 |  |  |  |  | 0.05 |  |  |  |  |
| N _AreaDept_ | 5 |  |  |  |  | 5 |  |  |  |  |
| Observations | 158 |  |  |  |  | 158 |  |  |  |  |
| Marginal R^2^ | .098 |  |  |  |  | .102 |  |  |  |  |
|  | *Positive Evading (Work)* | | | | | | | | | |
|  | *β* | *b* | *95% CI* | *SE* | *p* | *β* | *b* | *95% CI* | *SE* | *p* |
| (Intercept) | -0.06 | 3.39 | 2.09, 4.69 | 0.66 | <.001 | -0.06 | 3.69 | 1.85, 5.53 | 0.93 | <.001 |
| Age | 0.10 | 0.01 | -0.01, 0.03 | 0.01 | .208 | 0.08 | 0.01 | -0.01, 0.02 | 0.01 | .314 |
| Gender Male | -0.05 | -0.10 | -0.43, 0.23 | 0.17 | .535 | -0.03 | -0.07 | -0.40, 0.26 | 0.17 | .678 |
| Gender Prefer Not to Say | -0.08 | -0.87 | -2.59, 0.85 | 0.87 | .319 | -0.07 | -0.81 | -2.55, 0.93 | 0.88 | .360 |
| GIHS | 0.11 | 0.16 | -0.08, 0.40 | 0.12 | .201 |  |  |  |  |  |
| CIHS |  |  |  |  |  | 0.03 | 0.09 | -0.34, 0.52 | 0.22 | .670 |
|  |  |  |  |  |  |  |  |  |  |  |
| σ^2^ | 0.75 |  |  |  |  | 0.75 |  |  |  |  |
| τ_00_ _AreaDept_ | 0.02 |  |  |  |  | 0.02 |  |  |  |  |
| ICC | 0.03 |  |  |  |  | 0.03 |  |  |  |  |
| N _AreaDept_ | 5 |  |  |  |  | 5 |  |  |  |  |
| Observations | 158 |  |  |  |  | 158 |  |  |  |  |
| Marginal R^2^ | .023 |  |  |  |  | .014 |  |  |  |  |
|  | *Positive Opening (Work)* | | | | | | | | | |
|  | *β* | *b* | *95% CI* | *SE* | *p* | *β* | *b* | *95% CI* | *SE* | *p* |
| (Intercept) | -0.06 | 3.82 | 2.64, 5.00 | 0.60 | <.001 | -0.07 | 3.16 | 1.49, 4.83 | 0.85 | <.001 |
| Age | 0.02 | 0.00 | -0.01, 0.02 | 0.01 | .786 | -0.03 | -0.00 | -0.02, 0.01 | 0.01 | .660 |
| Gender Male | 0.02 | 0.03 | -0.27, 0.33 | 0.15 | .845 | 0.05 | 0.10 | -0.20, 0.39 | 0.15 | .508 |
| Gender Prefer Not to Say | -0.03 | -0.28 | -1.83, 1.28 | 0.79 | .727 | 0.01 | 0.06 | -1.52, 1.64 | 0.80 | .941 |
| GIHS | 0.27 | 0.37 | 0.15, 0.59 | 0.11 | .001 |  |  |  |  |  |
| CIHS |  |  |  |  |  | 0.23 | 0.58 | 0.19, 0.97 | 0.20 | .004 |
|  |  |  |  |  |  |  |  |  |  |  |
| σ^2^ | 0.61 |  |  |  |  | 0.62 |  |  |  |  |
| τ_00_ _AreaDept_ | 0.03 |  |  |  |  | 0.03 |  |  |  |  |
| ICC | 0.05 |  |  |  |  | 0.05 |  |  |  |  |
| N _AreaDept_ | 5 |  |  |  |  | 5 |  |  |  |  |
| Observations | 158 |  |  |  |  | 158 |  |  |  |  |
| Marginal R^2^ | .070 |  |  |  |  | .055 |  |  |  |  |
|  | *Positive Uniting (Work)* | | | | | | | | | |
|  | *β* | *b* | *95% CI* | *SE* | *p* | *β* | *b* | *95% CI* | *SE* | *p* |
| (Intercept) | -0.11 | 3.78 | 2.78, 4.78 | 0.51 | <.001 | -0.10 | 3.36 | 1.93, 4.79 | 0.72 | <.001 |
| Age | 0.04 | 0.00 | -0.01, 0.02 | 0.01 | .593 | -0.03 | -0.00 | -0.02, 0.01 | 0.01 | .669 |
| Gender Male | -0.04 | -0.06 | -0.31, 0.19 | 0.13 | .621 | 0.01 | 0.02 | -0.23, 0.27 | 0.13 | .858 |
| Gender Prefer Not to Say | 0.03 | 0.24 | -1.05, 1.53 | 0.65 | .714 | 0.07 | 0.59 | -0.76, 1.93 | 0.68 | .391 |
| GIHS | 0.36 | 0.43 | 0.25, 0.61 | 0.09 | <.001 |  |  |  |  |  |
| CIHS |  |  |  |  |  | 0.27 | 0.58 | 0.25, 0.91 | 0.17 | .001 |
|  |  |  |  |  |  |  |  |  |  |  |
| σ^2^ | 0.42 |  |  |  |  | 0.45 |  |  |  |  |
| τ_00_ _AreaDept_ | 0.04 |  |  |  |  | 0.04 |  |  |  |  |
| ICC | 0.09 |  |  |  |  | 0.09 |  |  |  |  |
| N _AreaDept_ | 5 |  |  |  |  | 5 |  |  |  |  |
| Observations | 158 |  |  |  |  | 158 |  |  |  |  |
| Marginal R^2^ | .117 |  |  |  |  | .068 |  |  |  |  |
|  | *Positive Composite (Work)* | | | | | | | | | |
|  | *β* | *b* | *95% CI* | *SE* | *p* | *β* | *b* | *95% CI* | *SE* | *p* |
| (Intercept) | -0.13 | 3.61 | 2.70, 4.51 | 0.46 | <.001 | -0.12 | 3.12 | 1.83, 4.41 | 0.65 | <.001 |
| Age | 0.07 | 0.01 | -0.01, 0.02 | 0.01 | .336 | 0.01 | 0.00 | -0.01, 0.01 | 0.01 | .943 |
| Gender Male | -0.03 | -0.04 | -0.27, 0.18 | 0.11 | .715 | 0.02 | 0.03 | -0.20, 0.25 | 0.11 | .807 |
| Gender Prefer Not to Say | -0.00 | -0.00 | -1.18, 1.17 | 0.60 | .997 | 0.04 | 0.31 | -0.90, 1.51 | 0.61 | .618 |
| GIHS | 0.33 | 0.36 | 0.19, 0.53 | 0.08 | <.001 |  |  |  |  |  |
| CIHS |  |  |  |  |  | 0.26 | 0.52 | 0.22, 0.82 | 0.15 | .001 |
|  |  |  |  |  |  |  |  |  |  |  |
| σ^2^ | 0.35 |  |  |  |  | 0.36 |  |  |  |  |
| τ_00_ _AreaDept_ | 0.04 |  |  |  |  | 0.04 |  |  |  |  |
| ICC | 0.10 |  |  |  |  | 0.10 |  |  |  |  |
| N _AreaDept_ | 5 |  |  |  |  | 5 |  |  |  |  |
| Observations | 158 |  |  |  |  | 158 |  |  |  |  |
| Marginal R^2^ | .100 |  |  |  |  | .066 |  |  |  |  |
|  | *Negative Attacking (Work)* | | | | | | | | | |
|  | *β* | *b* | *95% CI* | *SE* | *p* | *β* | *b* | *95% CI* | *SE* | *p* |
| (Intercept) | 0.00 | 2.72 | 1.60, 3.84 | 0.57 | <.001 | -0.00 | 5.14 | 3.64, 6.63 | 0.76 | <.001 |
| Age | -0.01 | -0.00 | -0.01, 0.01 | 0.01 | .947 | 0.03 | 0.00 | -0.01, 0.02 | 0.01 | .677 |
| Gender Male | 0.04 | 0.08 | -0.21, 0.37 | 0.15 | .603 | 0.03 | 0.06 | -0.21, 0.33 | 0.14 | .663 |
| Gender Prefer Not to Say | 0.16 | 1.54 | 0.04, 3.05 | 0.76 | .045 | 0.11 | 1.09 | -0.34, 2.52 | 0.73 | .135 |
| GIHS | -0.15 | -0.19 | -0.40, 0.02 | 0.11 | .070 |  |  |  |  |  |
| CIHS |  |  |  |  |  | -0.36 | -0.85 | -1.20, -0.49 | 0.18 | <.001 |
|  |  |  |  |  |  |  |  |  |  |  |
| Observations | 158 |  |  |  |  | 158 |  |  |  |  |
| R^2^ | .047 |  |  |  |  | .151 |  |  |  |  |
|  | *Negative Evading (Work)* | | | | | | | | | |
|  | *β* | *b* | *95% CI* | *SE* | *p* | *β* | *b* | *95% CI* | *SE* | *p* |
| (Intercept) | 0.04 | 3.77 | 2.13, 5.41 | 0.83 | <.001 | 0.03 | 6.28 | 4.04, 8.52 | 1.13 | <.001 |
| Age | 0.03 | 0.00 | -0.02, 0.02 | 0.01 | .718 | 0.04 | 0.01 | -0.01, 0.02 | 0.01 | .596 |
| Gender Male | -0.04 | -0.11 | -0.53, 0.30 | 0.21 | .590 | -0.05 | -0.12 | -0.52, 0.28 | 0.20 | .555 |
| Gender Prefer Not to Say | 0.08 | 1.13 | -1.03, 3.30 | 1.10 | .303 | 0.05 | 0.71 | -1.42, 2.85 | 1.08 | .509 |
| GIHS | -0.07 | -0.13 | -0.43, 0.17 | 0.15 | .398 |  |  |  |  |  |
| CIHS |  |  |  |  |  | -0.24 | -0.79 | -1.32, -0.27 | 0.27 | .003 |
|  |  |  |  |  |  |  |  |  |  |  |
| σ^2^ | 1.18 |  |  |  |  | 1.13 |  |  |  |  |
| τ_00_ _AreaDept_ | 0.04 |  |  |  |  | 0.02 |  |  |  |  |
| ICC | 0.03 |  |  |  |  | 0.02 |  |  |  |  |
| N _AreaDept_ | 5 |  |  |  |  | 5 |  |  |  |  |
| Observations | 158 |  |  |  |  | 158 |  |  |  |  |
| Marginal R^2^ | .016 |  |  |  |  | .064 |  |  |  |  |
|  | *Negative Composite (Work)* | | | | | | | | | |
|  | *β* | *b* | *95% CI* | *SE* | *p* | *β* | *b* | *95% CI* | *SE* | *p* |
| (Intercept) | 0.00 | 3.13 | 2.07, 4.18 | 0.54 | <.001 | 0.00 | 5.57 | 4.17, 6.97 | 0.71 | <.001 |
| Age | 0.00 | 0.00 | -0.01, 0.01 | 0.01 | .984 | 0.04 | 0.00 | -0.01, 0.02 | 0.01 | .617 |
| Gender Male | 0.01 | 0.01 | -0.26, 0.29 | 0.14 | .924 | -0.00 | -0.00 | -0.25, 0.25 | 0.13 | .989 |
| Gender Prefer Not to Say | 0.15 | 1.41 | 0.01, 2.83 | 0.72 | .052 | 0.11 | 0.97 | -0.37. 2.31 | 0.68 | .155 |
| GIHS | -0.15 | -0.18 | -0.38, 0.02 | 0.10 | .072 |  |  |  |  |  |
| CIHS |  |  |  |  |  | -0.38 | -0.84 | -1.17, -0.51 | 0.17 | <.001 |
|  |  |  |  |  |  |  |  |  |  |  |
| σ^2^ | 0.51 |  |  |  |  | — |  |  |  |  |
| τ_00_ _AreaDept_ | 0.00 |  |  |  |  | — |  |  |  |  |
| ICC | 0.00 |  |  |  |  | — |  |  |  |  |
| N _AreaDept_ | 5 |  |  |  |  | — |  |  |  |  |
| Observations | 158 |  |  |  |  | 158 |  |  |  |  |
| Marginal R^2^ | .045 |  |  |  |  | .164 |  |  |  |  |

**References**

Coleman, P. T., & Lim, Y. Y. J. (2001). A systematic approach to evaluating the effects of collaborative negotiation training on individuals and groups. *Negotiation Journal*, 17(4), 363-392.
